# Clues to Long COVID Linked to Virulence and Infectivity Found in Shell Proteins

**DOI:** 10.3390/arm94020018

**Published:** 2026-03-11

**Authors:** Gerard Kian-Meng Goh, James A. Foster, Vladimir N. Uversky

**Affiliations:** 1 Goh’s BioComputing, Singapore 548957, Singapore; 2 Department of Biological Sciences, University of Idaho, Moscow, ID 83844, USA; 3 Institute for Bioinformatics and Evolutionary Studies, University of Idaho, Moscow, ID 83844, USA; 4 Department of Molecular Medicine, Morsani College of Medicine, University of South Florida, Tampa, FL 33612, USA

**Keywords:** coronavirus, COVID-19, intrinsic disorder, membrane, nucleocapsid, nucleoprotein, Omicron, pangolin, shell, virulence, long COVID, attenuation, variant, immune, perforin, complement system, macrophage, infectivity, pathogenesis, reservoir, artificial intelligence, AI, hard shell, lysosome, unstructured, NL63, spike, pathogenesis, phagocytosis, contagiousness, dendritic, phagocyte, monocyte, neutrophil

## Abstract

**Highlights:**

**What are the main findings?**

- The abnormally hard M, detected among all SARS-CoV-2 viruses using AI, is believed to be the cause of SARS-CoV-2 high infectivity as it is more resistance to salivary and mucosal antimicrobial enzymes and, thereby, forces the infected person to shed much greater quantities of viral particles.
- N disorder could modulate the severity of COVID-19 and long COVID by allowing faster replication of the virus as correlations between the inner shell (N) disorder and virulence have been found.

**What are the implications of the main findings?**

- Current knowledge of physiology, immunology and biochemistry suggests that the unusually hard M is not just associated with infectivity but also long COVID as the virus could resist the antimicrobial enzymes in the phagocyte and is thus able to dwell in it, which could become a virus reservoir.
- An understanding of the mechanisms by which the virus hides in the body, could lead to better treatments of long COVID by targeting the reservoir via antiviral drugs or correctly timed vaccination using the appropriate vaccine version.

**Abstract:**

Clinical, experimental, and computational evidence of COVID-19 virulence and infectivity has been linked to SARS-CoV-2 shell disorder. A strong link was first discovered using an AI disorder-predicting tool, which detected an unusually hard (low disorder) outer shell among all SARS-CoV-2-related viruses but not in the 2003 SARS-CoV-1. This could account for the high infectivity found in SARS-CoV-2—but not in SARS-CoV-1—as it is believed that hard shells protect viral particles from the onslaught of the antimicrobial enzymes present in the respiratory system and saliva. As a result, much larger quantities of particles are shed by COVID-19 patients. Abnormally hard outer shells (M) are associated with burrowing animals, e.g., pangolins, and SARS-CoV-2 likely acquired these shells due to its long-term evolutionary interactions with pangolins. As for virulence, the inner shell of SARS-CoV-2 (N) has been found to exhibit lower disorder than that of SARS-CoV-1. This lower disorder is consistent with the fact that SARS-CoV-2 is less virulent than SARS-CoV-1, as higher disorder in the inner shell is associated with more efficient protein–protein binding during replication. The link between N/M disorder and virulence or infectivity falls under the umbrella of shell disorder models (SDMs), which can connect virulence, infectivity, and long COVID under one coherent concept. Evidence of the reliability and reproducibility of SDMs as applied to COVID-19 is examined. The hard M that is resisting the antimicrobial enzymes in the respiratory system can be extended to immunological enzymes, especially those found in phagocytes such as macrophages, which can therefore become a reservoir for the virus.

## 1. Introduction

### 1.1. Goals and Overview

As COVID-19 (coronavirus disease 2019) [1,2,3] has already become endemic, many questions remain not fully answered. These include enigmas pertaining to infectivity, virulence, and long COVID, such as the following: What are the underlying causes of infectivity, virulence, and long COVID? How are they linked together? Why is long COVID manifested differently from long SARS (2003 SARS-CoV-1)? What is the source of the virus reservoir in long COVID? This review focuses on the application of the concept of intrinsic protein disorder to COVID-19 and, more specifically, on three closely related models—SDMs (shell disorder models)—that use AI to detect protein disorder, encompassing a variety of viruses [4]. The models link shell disorder to infectivity, virulence, and, potentially, long COVID coherently under one concept. SDMs examine the effects of intrinsic protein disorder with respect to shell proteins, i.e., M (outer shell protein) [4,5] and N (inner shell protein) [4], in the case of SARS-CoV01/2, investigating the behavior of the virus and its manifestation. While greater N disorder affects the infectivity of the virus by producing more viral particles as greater disorder allows more efficient protein–protein binding, a peculiar phenomenon is detected in all SARS-CoV-2-related viruses, but not SARS-CoV-1. They have an unusually hard outer shell (M) that is seldom seen among CoVs, except those associated with a burrowing animal, such as rabbits and pangolins. This observation leads to the belief that COVID-19 patients can shed much larger quantities of viral particles despite SARS-CoV-2 having lower N disorder than SARS-CoV-1. SARS-CoV-2 appears to achieve this by having a harder M (lower M disorder) that allows greater viral shedding due to its ability to resist the onslaught of antimicrobial enzymes found in the respiratory system and saliva. This paradigm has important implications for long COVID since many of the immunologically related enzymes—especially those found in phagocytes, such as macrophages—are involved in similar chemical mechanisms as those of the respiratory and salivary enzymes. This could provide clues that could help resolve the ongoing mystery in the search for the source of a virus reservoir in long COVID. The case for SDMs as applied to M and N is presented. Computational, experimental, and clinical evidence is examined, with an emphasis on reproducibility and reliability.

### 1.2. Pangolin Footprints: Enigmas of COVID-19-Related Viruses

It has been more than 4 years since the first outbreak of COVID-19 [1,2,3]. Even as we are only beginning to understand COVID-19 uncertainties, there is still much to be uncovered and resolved. For instance, why is SARS-CoV-2 highly contagious in contrast to 2003 SARS-CoV (SARS-CoV-1, SARS: severe acute respiratory syndrome; CoV: coronavirus)? The total number of people infected thus far is more than 700 million [3], whereas there were only about 10,000 cases in the 2002–2003 SARS outbreak [4]. Why is SARS-Co-2 less virulent and much more infectious than SARS-CoV-1? What are the structural differences responsible for the differences? What is the cause of long COVID? Early in the outbreak, it was believed that the reason for the extraordinary contagiousness of COVID-19 lies solely in the spike (S) protein [6,7,8,9,10,11,12]. This study re-examines the roles of two highly important, although less-researched, proteins—M and N—while keeping the functions of S in mind.

The framework of many studies was established using an AI tool to study the sequence of the M and N proteins. One important but peculiar discovery using the protein disorder AI tool PONDR^®^-VLXT [13,14,15,16,17] is that all SARS-CoV-2-related viruses—not only SARS-CoV-2—have among the hardest outer shells (low M disorder) known within the CoV family [18,19,20,21]. It is believed that it is this anomaly pertaining to M that is primarily responsible for the high contagiousness of COVID-19, since a harder M provides greater resistance for the virus against the large array of antimicrobial enzymes present in the saliva and mucus [21,22,23,24,25,26,27,28], thus making it more likely for the host to shed much more infectious particles [29]. N, on the other hand, could help modulate infectivity and virulence, as greater N disorder allows for more efficient protein–protein/RNA/lipid binding [4,16,30,31,32,33], which could result in the more rapid replication of the virus, especially in vital organs [34,35,36,37,38,39,40].

A retrospective search after the initial COVID-19 outbreak yielded a bat-CoV sample (RaTG13) obtained from a Yunnan cave in 2013 that shared 96.4% genetic identity with SARS-CoV-2 [4,5,6,7,8,9,10,11,12,13,14,15,16,17,18,19,20,21,22,23,24,25,26,27,28,29,30,31,32,33,34,35,36,37,38,39,40,41,42,43]. Furthermore, two sets of pangolin-CoVs—obtained from pangolins confiscated by customs in Guangxi (GX) and Guangdong (GD) provinces during 2017–2018 (Pang2017 and Pang2018) and 2019 (Pang2019), respectively—have approximately 90% genetic proximity to SARS-CoV-2 [44,45,46,47,48]. Later, pangolin samples obtained in Vietnam exhibited similar results [49]. Likewise, a series of COVID-19-related bat-CoVs (BANAL) were found in Laos [50,51]. In fact, one of the samples, BANAL-52, exhibited even greater genetic proximity (96.8%) to SARS-CoV-2 than that observed between RaTG13 and SARS-CoV-2.

A search for similarly hard M yielded CoVs associated with burrowing animals, such as rabbits. This enigmatic association explains the true intimate relationship between all COVID-19-related viruses and pangolin-CoVs since pangolins are also burrowing animals [4,18,19,20,52,53,54]. The hard outer shell (M) is necessary as the virus must be able to survive longer in buried feces before further transmission.

While most scientists believe that COVID-19 is the result of a zoonotic spillover, a better understanding of its evolution helps. The knowledge of N and M proteins can render our understanding of this evolution more complete, as it bridges the relationship between SARS-CoV-2 and pangolin-CoV. The relationship, as evidenced by the pangolin’s molecular “footprints”, can account for COVID-19 behavior. The implications of this relationship will be revisited in greater detail later.

One odd characteristic found in all SARS-CoV-2-related viruses and their variants found thus far is the unusually hard outer shell (low M disorder), which is typically found only in CoV associated with a burrowing animal. This hallmark is one of the “pangolin footprints” found in all COVID-19-related viruses discovered so far. The other set of footprints involves attenuations associated with the lower N disorder observed in Pang2017 and later in the Omicron variant [18,19,20,52,53,54,55,56]. The harder inner shell (N), especially in Pang2017, may also reflect N’s ability to protect viral RNA in buried feces, similarly to M [52,55].

Evidence of pangolin footprints offers clues that are beginning to uncover many of the COVID-19 mysteries that are still haunting us. These include questions such as the following: why is SARS-CoV-2 substantially more infectious than SARS-CoV-1? Why is COVID-19 highly infectious to this day? Why is SARS-CoV-2 less virulent than SARS-CoV-1? Why is Wuhan-Hu-1 much more virulent than Omicron? What is the actual structural cause of long COVID? Many of these questions have remained unanswered, but the study of the M and N proteins is now beginning to provide some answers from one integrated concept, namely, pangolin footprints. We should not be at all surprised by the explanatory prowess of the N and M proteins, as they are the most abundant proteins found in the cell and virion, respectively.

### 1.3. Long COVID: A Mystery

Another major COVID-19 mystery is the presence of long COVID among many patients, i.e., the persistence of symptoms long after recovery [57,58]. Even as progress has been made in research to explain the mechanism and cause of long COVID, it has remained, by and large, a mystery [59,60,61,62,63,64], especially with regard to the source of a reservoir, if any. In this study, we review a new and novel explanation. This explanation involves the unusually hard M found in SARS-CoV-2 that allows the virus to resist immune enzymes and thus enables the virus to hide in phagocytes. The abnormally hard outer shell M—which protects the virion from the onslaught of antimicrobial enzymes found in saliva and mucus [19,20,21,22,23,24,25,26,27,28,40,52]—is also likely to reduce the chances of the elimination of viral particles via actions of the immune system. We examine the literature pertaining to current immunological knowledge that could provide a potential framework for the mechanism of long COVID caused by hard M. SDMs, and thus, the concept of intrinsic protein disorder will be heavily used to gain insight into the potential mechanisms involved.

The abnormally hard outer shell (M), which protects the virion from the onslaught of antimicrobial enzymes found in saliva and mucus [19,20,21,22,23,24,25,26,27,28,40,52], is also likely to reduce the chances of viral particle elimination via actions of the immune system. Upon the examination of immunological principles, shell disorder models (SDMs) further pinpoint the exact immunological mechanisms that are likely to be hindered.

### 1.4. Varying Strengths in Evidence Presented

SDMs provide a coherent framework that links infectivity, virulence, and long COVID. Evidence for each COVID-19 aspect will be introduced. An overview of comparative studies of the infectivity and virulence of some CoVs and various SARS-CoV-2 variants—including SARS-CoV-1, bat/pangolin-CoVs, SARS-CoV-2 variants (e.g., Wuhan, Hu-1, Omicron), and NL63—will involve experimental, computational, theoretical, and clinical data. Overall, we observe that, by and large, SDMs consistently and accurately predict the levels of infectiousness and virulence of SARS-CoV-1/2 and their variants based on the measured predicted disorder (PID: percentage of intrinsic disorder). Furthermore, the disorder of M and N can provide a coherent molecular and physiological explanation for virulence, infectivity, and long COVID. The measurement of virulence can be somewhat problematic, as the precision of CFR (case fatality rate) is often questioned, even if it can highlight the difference in virulence between SARS-CoV-1 (CFR ~10%) and SARS-CoV-2 (Wuhan-Hu-1 CFR ~2%) [1,2,3,4]. To mitigate this problem, experimental data involving viral titers and cell plaques are also examined [55]. The infectiousness of SARS-CoV-1/2 and its variants is more easily observed clinically by noting the number of infected patients in an area within a period of time. SARS-CoV-2 is, of course, substantially more infectious than SARS-CoV-1 given the number of people each has infected. Clinical studies have shown that SARS-CoV-2 patients shed significantly more viral particles [29]. Again, SDMs put forth a novel explanation (i.e., a hard M that is resistant to antimicrobial enzymes in saliva and mucus) that is consistent with our current knowledge of biochemistry, physiology, and virology [18,19,20,21,22,38,39,40,41]. Similarly, such reproducibility is reflected in SDMs’ explanation of the observation that SARS-CoV-1 is more virulent than SARS-CoV-2, whereas Omicron is less virulent than all previous variants. It should be noted that many of the predictions made are not restricted to CoVs but also apply to a variety of other viruses, including Nipah virus, Ebola virus, and dengue virus (DENV) [34,35,36,37,38,39,40], as discussed below.

COVID-19 enigmas can be broadly categorized according to three aspects: infectivity, virulence, and long COVID. Further signs of the reliability of SDMs can be observed when they explain each aspect of the disease in connection with other features in terms of physiology, virology, and biochemistry. It must be kept in mind that the strength of SDMs in terms of reliability and reproducibility is dependent not only on the individual explanation of each aspect of COVID-19 but also on how each aspect is interwoven with the others according to the explanations. Nevertheless, we need to understand that even as all explanations presented in this review offer clues for further investigation, they are of varying levels of strength. COVID-19 virulence and infectivity have been examined and investigated more intensively since the outbreak of the pandemic, and the results of related experimental and clinical data have been carefully studied, even as more direct studies would certainly help. The application of SDMs to long COVID, on the other hand, is still in its infancy, as insufficient attention and resources have been directed to this topic in the past. Therefore, further research is needed on this issue, both by our group and other independent groups. We believe and argue, however, that there is ample evidence—based on research into the mechanisms of COVID-19 virulence and infectivity and our current knowledge of physiology and immunology—to provide a plausible and solid theoretical basis for the role of shell disorder (or the lack of disorder) in long COVID. As we will see, the virus is detected in the blood and throughout the body of long COVID patients; therefore, this offers some support to our suggestions related to the mechanism of long COVID.

While the individual SDM evidence for each aspect is of varying concreteness, the reproducibility of a model should be evaluated based on how coherent and consistent its explanations are with respect to the central concept, which, in this case, is intrinsic protein disorder (or the lack of disorder) in viral shell proteins. It should also be kept in mind that even as phylogenetic evidence is introduced in this study to explain the crucial role of pangolins in the evolution of SARS-CoV-2—which accounts for its peculiar characteristics—evolutionary and phylogenetic studies, such as what is being presented here, usually leave plenty of room for debates. Such analysis is therefore presented as clues towards further research.

## 2. The Shell Disorder Models (SDMs): Three Closely Related Models

### Three Closely Interrelated Models

**Figure 1:**
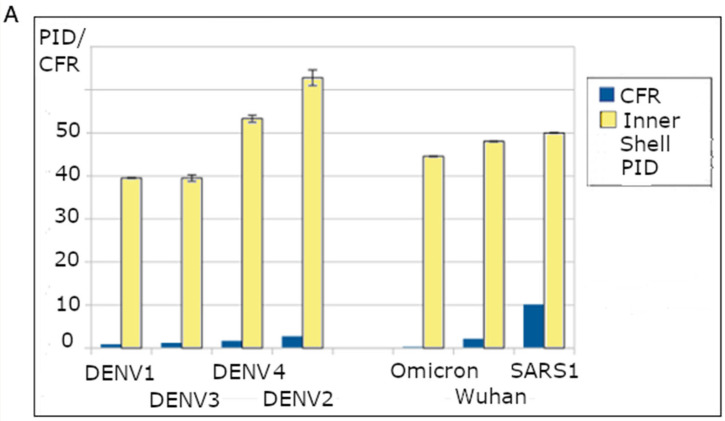

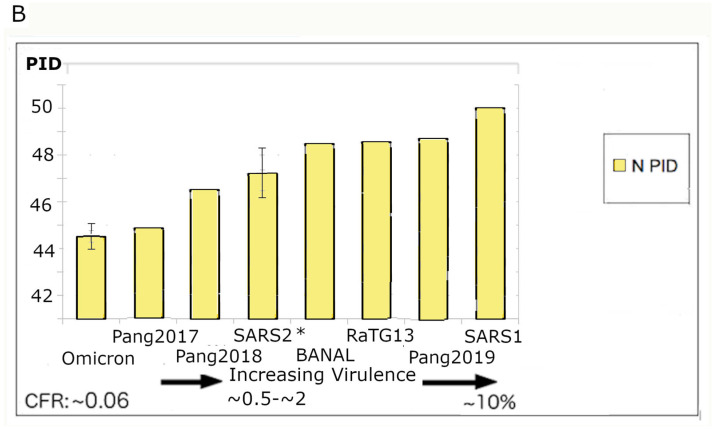
Virulence for inner SDM (shell disorder model) [40]. (**A**) Shells PID of DENV and SARS-CoV-2. The inner shell of DENV has been found to be correlated with virulence (r = 0.95) [34,35]. (**B**) Correlation between the SARS-related viruses and N PID. The correlation is based on the estimated CFRs of SARS-CoV-1/2 and Omicron. Note: DENV and SARS-CoV-1/2 shells are not correlated. They are placed together for illustrative purposes only. SARS2* refers to the Wuhan-Hu-1 strain (wild-type).

Three closely related models were developed using the concept of intrinsic protein disorder via AI tools. Intrinsic protein disorder refers to the lack of structure in an entire protein or part of a protein. Disorder is known to have various important functions, especially in protein–protein/RNA/DNA/lipid/carbohydrate binding, and various tools have been developed to recognize disorder regions and disordered proteins. Among the first developed is PONDR^®^-VLXT, which involves the use of neural network AI to recognize disordered residues given the sequence input [13,14,15,16,17]. PONDR^®^-VLXT has been shown to be a highly appropriate tool, particularly when it involves viral proteins of a large variety of viruses, including Ebola virus (EBOV), dengue virus (DENV), Nipah virus, and HIV [4,18,19,20,34,35,36,37,38,39,40,52,53,54,55,56,65,66,67]. PONDR^®^-VLXT is especially suited for the study of viral structural proteins, as it is highly sensitive in the detection of disorder in structured proteins [16]. The first SDM was initiated before 2008 when PONDR^®^-VLXT was used to examine the shell proteins of a variety of viruses. A useful number used is the percentage of intrinsic disorder (PID), which is defined as the number of predicted disordered residues divided by the total number of residues in a protein chain [4,65]. A disordered residue is predicted to be disordered if its VLXT score is 0.5 or above. Upon comparisons of the shell disorder of a fairly large number of viruses, it became obvious that the outer shell of HIV, especially HIV-1, exhibits an abnormally high average disorder. It was also discovered that HSV and HCV share this similar characteristic, even though it is not as pronounced as in HIV-1 [4,18,19,20,34,35,36,37,38,39,40,65,66,67]. Since very few other viruses, if any, have this characteristic, it seems to be related to the ability of the viruses to evade the immune system, resulting in the absence of effective vaccines for the mentioned viruses. This SDM was labeled “Viral Shapeshifting” [4] and became the parent model for two other closely related SDMs, as observed in Figure 1. Given the behaviors of HIV, HCV, and HSV, higher disorder in the outer shell can also be associated with the ability to penetrate hard-to-reach places such as the brain and placenta, as in the case of the Zika virus [34,35]. Protein disorder enhances the efficiency of protein–protein/DNA/RNA/lipid/glycoprotein binding [16,30,31,32,33,55,58].

Another model was developed and published in 2015 when it was discovered that there is a strong correlation between DENV virulence and disorder at the inner shell protein [34,35]. Similar correlations were found in a large variety of other viruses, including NIV, CoVs, and EBOV [4,34,35,36,37,38,39,40,52,53,54,55,56,65,66,67] (it must be noted that while Figure 1 provides examples from CoVs and DENV, the SDM is by no means restricted to these viruses). The presence of such correlations is related to the fact that the inner shells are often associated with replication in many viruses and because disorder provides for greater efficiency in protein–protein/RNA/DNA/lipid/glycoprotein binding [16,30,31,32,33]. These form the basis of the virulence–inner shell disorder model (Table 1).

**Figure 2:**
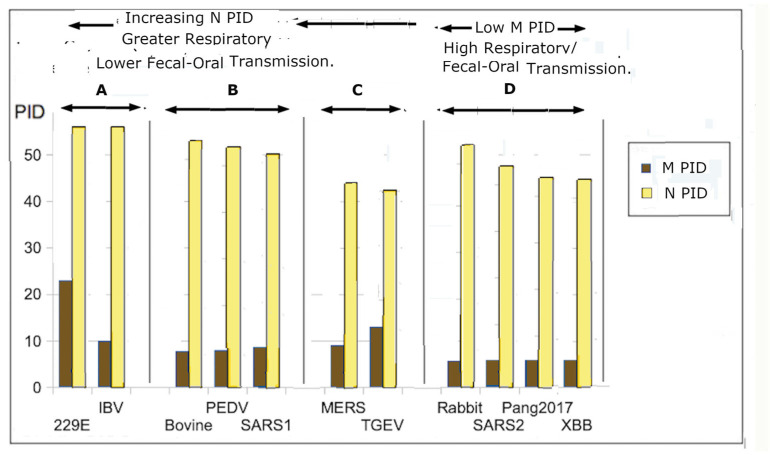
CoV-transmission shell disorder model (SDM) CoVs [55]. In groups A–C, the levels of respiratory/fecal–oral transmission are heavily dependent on N PID, whereas those in group D have unusually low M PIDs (M disorder) that are usually associated with burrowing animals such as pangolins. Group D includes all COVID-19-related viruses (multivariate analysis: *p* < 0.001, r ~0.8, N = 32).

The third SDM was first published in 2012 [53] before MERS-CoV was discovered in 2013 [55]. The model divided CoVs into three groups, labeled A–C, which can be observed in Figure 2, with group D added during the COVID-19 pandemic [18,19,20,40,53,55,56]. Group D was not recognized in the initial model because there were very few CoVs that involved burrowing animals such as rabbits and pangolins at the time [4,16,17,18,52,53]. Before the COVID-19 pandemic, nearly all CoVs available in our database had M PIDs of at least 8% (see Figure 2). The exceptions are the few available CoVs associated with burrowing animals. It was only during the pandemic that it was discovered that all SARS-CoV-2-related viruses have M PIDs lower than 7% (4–6.3%) (see Figure 2 and Table 2). While the statistical differences appear small, in reality, they are not insignificant or trivial, as we will observe later in the case of M, which is the most abundant protein that encases the entire virion. Even small changes will affect the rigidity of the entire shell. This CoV-transmission SDM invokes the same molecular principle that the virulence–inner SDM uses, which involves greater disorder in the inner shell that provides greater efficiency in viral replication. However, the CoV-transmission SDM extends this principle to the fecal–oral and respiratory transmission potentials, showing that respiratory transmission is viable only when sufficient copies of the virus are shed nasally. This results in N being adequately disordered. Multivariate analysis has found a strong correlation between modes of transmission and levels of M/N PIDs, with statistical significance (multivariate analysis: *p* < 0.001, r ~0.8, N = 32).

The CoV-transmission SDM categories with respect to SARS-CoV-1 in group B consist of CoVs with intermediate fecal–oral and respiratory transmission potentials. Upon the publication of the original manuscript, the MERS-CoV outbreak took place in 2012–2013, when the SDM had to place MERS-CoV in group C, in which the CoVs exhibited higher and lower fecal–oral and respiratory transmission potentials [54]. This prediction has been reproduced clinically and experimentally [4]. It is also known that MERS-CoV has long been entrenched among camels, especially farmed camels, where it spreads easily by fecal–oral means.

## 3. Pangolin Molecular Footprint as an Evolutionary Link to the Peculiar Characteristics Found in SARS-CoV-2

### 3.1. The First Sign of a Pangolin Footprint: Abnormally Hard Shells in All SARS-CoV-2-Related Viruses

When the CoV-transmission SDM was first developed and implemented in 2012–2013, a strong correlation was observed between N disorder (N PID) and the modes of transmission [4,53,54]. However, statistical calculations detected a small correlation between M disorder (M PID) and the modes of transmission. PID is defined as the number of disordered residues divided by the total number of residues in the protein. Previously, it was not understood why such a correlation existed. It was not until the arrival of the COVID-19 pandemic, accompanied by a flood of data, that pieces began to fall in place. The CoV-transmission SDM was applied to Wuhan-Hu-1 as soon as the M and N proteins became available, with Wuhan-Hu-1 exhibiting N and M PIDs of 48.2% and 5.9%, respectively [14,40,52,53,54,55]. Using the original model, the SDM placed SARS-CoV-2 in group B, which is the same group as SARS-CoV-1.

The SDM identified a highly unusual feature of the virus that was seldom observed in our curated database of known CoVs: The outer shell of SARS-CoV-2 is abnormally hard, i.e., it has a low M PID [18,19,20]. As more data became available, it became clear that the odd hard outer shell was not a feature exclusive to SARS-CoV-2, but it was also observed in all COVID-19-related viruses, as seen in Table 2 and Figure 2. We can observe that the hard M (low M PID < 7%, Table 2) extends to all SARS-CoV-2-related viruses, including pangolin-CoVs and bat-CoVs such as RaTG13 and the Laotian bat-CoV (BANAL). The details found in the tables are also provided in previous studies [52,55,56], and the protein sequences were obtained either from UniProt [68] or NCBI-Protein [69]. The PONDR^®^-VLXT scores were obtained by inputting the sequences into PONDR^®^-VLXT [13,14,15,16,17]. The PIDs (percentages of intrinsic disorder) were calculated as the number of disordered residues divided by the total number of proteins in a protein chain [4,65,66,67].

It was only after the pandemic—which supplied new data—that we recognized the need to add group D, and we understood why group C had initially been overlooked. A retrospective search for similarly low M PIDs can only be found in CoVs associated with burrowing animals such as rabbits [18,19,55]. Because there were very few such CoVs in the public database before the pandemic, the CoV-transmission SDM was inevitably incomplete at that time. According to our previously published studies [19,20], it also became obvious that the hard M was responsible for the high transmissibility among humans by protecting the virus from the myriad of antimicrobial enzymes found in the mucus and saliva [18,19,20,40,45,52]. As a result, the host will shed large amounts of infectious particles nasally and orally. There is also reason to suspect that the abnormally hard M may play a significant role in long COVID [40], as the host immune system may often have difficulties when destroying and eliminating viral particles.

### 3.2. Signs of Attenuation: Another Pangolin Footprint

The three SDMs present us with a set of tools that can output a wide range of specific predictions with respect to SARS-CoV-2 and its closely related relatives. Similarly to how SDM’s prediction was applied to MERS, the predictions have exhibited consistency when applied to experimental and clinical data. Figure 2 summarizes an example of the application of the virulence–inner SDM to COVID-19 that is consistent with clinical and experimental data. A strong correlation can be found between COVID-19 virulence and N PID. Using the case fatality rate (CFR) as a benchmark for virulence, we can see that the CFR varies with the N disorder (N PID), with SARS-CoV-1 having the highest CFR of about 10%, followed by the non-Omicron SARS-CoV-2 variants and then Omicron (Figure 1B). Even without using CFR, the virulence–inner SDM makes specific predictions about the levels of virulence of SARS-related viruses and SARS-CoV-1. SARS-CoV-1 is predicted to be of higher virulence given its N PID of 50%, followed by Wuhan-Hu-1 (N PID: 48.2%) and the other non-Omicron variants (Delta N PID ~47.1 + 0.5%). Omicron (N PID ~44.5 + 0.4%) is the least virulent thus far [40,52,55,56]. It should also be known that CFR is just one measure of the link. As we shall observe, it has been complemented with viral titer and cell/tissue studies. The SDMs can pinpoint mechanisms that account for the predictions. In the context of COVID-19 virulence, higher N disorder corresponds to greater viral replication efficiency, as N is intimately involved in the replication process and increased disorder facilitates more effective protein–protein/RNA/DNA/lipid/glycoprotein interactions.

### 3.3. Pangolin Footprints: Implications and Manifestations

Two important predictions (shown in Figure 1B) that have been reproduced are that Omicron and Pangolin-2017 (Pang2017) are attenuated based on their low N PIDs. This prediction was first mentioned in a publication in 2020 [14] and later reproduced in at least two laboratories. Omicron, however, did not emerge until November 2021. When the first Omicron sequence became available, it was found that the Omicron subvariant, BA1, has an N of 44.7%, which is very close to that of Pang2017 at 44.8% [20,52,55,56]. Similarly to how Pang2017 was predicted to be attenuated, Omicron or, at least, BA1 must be considered attenuated. It was later shown that all Omicron subvariants have similar N PIDs, with the later ones exhibiting even lower values (XBB.1.16, N PID: 44.2%). Both Pang2017 and Omicron have been clinically and experimentally shown to be attenuated with lower viral titers [40,51,70,71,72,73,74,75]. Interestingly, in contrast, Pang2019’s N PID resembles that of non-Omicron variants, and not coincidentally, its virulence and viral titers do not resemble those of Omicron or Pang2017 [76,77,78].

### 3.4. Implications and Manifestations: Infectivity and Virulence

**Figure 3:**
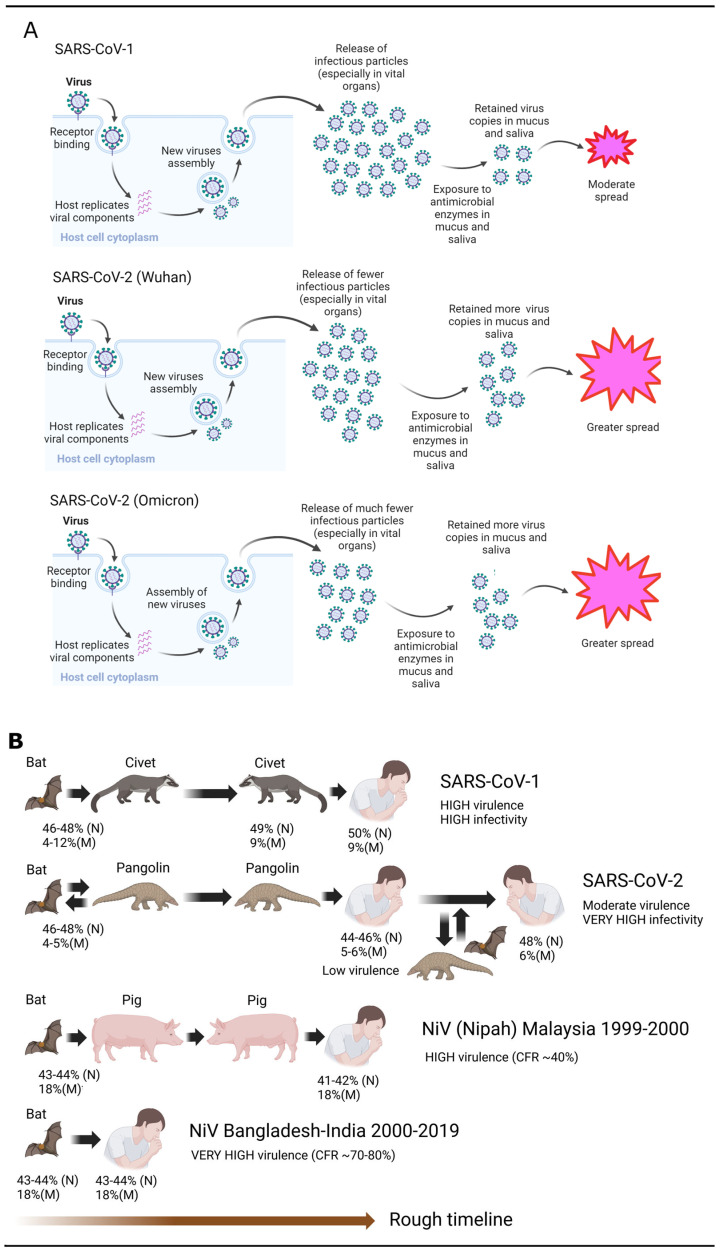
Effects of M and N disorder on virulence and infectivity [55]. (**A**) Effects of M and N disorder on virus replication. (**B**) Effects of the environment on virulence. SARS-CoV-1 produces high quantities of viral particles, especially in vital organs, because of its high N disorder, but by the time they reach the nasal and oral cavity, many have already been eliminated by mucosal and salivary antimicrobial enzymes as a result of its lower M PID. SARS-CoV-2, on the other hand, produces fewer particles in the entire respiratory system, but more particles can resist the antimicrobial enzymes because of their hard M; therefore, its patients shed more particles. NiV is highly virulent when it is spread directly from bats, whereas the variant with pigs as an intermediate host was less virulent. The inner shell disorder of the latter was observed. Similar observations were made for SARS-CoV-1/2.

Figure 3A summarizes the implications of SDMs and the effects of different N and M PIDs on the manifestation of COVID-19. SARS-CoV-1 (SARS1) has a higher N PID (~50%) and produces more viral particles, especially in the vital organs. However, because its M is not as hard (M PID ~9%) [4], it offers less resistance to the onslaught of salivary and mucosal antimicrobial enzymes and produces sufficient infectious particles for viable respiratory transmission. However, SARS-CoV-2 presents a different story. The Wuhan-Hu1 strain exhibits slightly lower N disorder (N PID ~48%) but a substantially lower M disorder (M PID ~5.7%) [40]. According to SDMs, the virus will replicate well even in vital organs but not as efficiently as SARS-CoV-1. Even though Wuhan-Hu-1 does not replicate in large quantities in contrast to SARS-CoV-1, the patient sheds more infectious particles because the harder M offers more resistance to the antimicrobial enzymes in the saliva and mucus [40,52]. For this reason, Wuhan-Hu-1 is virulent, but not to the degree of SARS-CoV-1. On the other hand, it is substantially more infectious than the latter due to the immense amount of shedding. Omicron presents a different scenario with a similarly low M PID (~5.7%) but much lower N PID (~44.5%). With respect to these, the SDMs predict low virulence but high infectivity, even if lower than Wuhan-Hu-1. All these predictions have been reproduced experimentally and clinically [52,55,70,71,72,73,74,75,76,77,78,79,80,81].

Figure 3A describes the underlying mechanism of how differences in N and M disorder affect the manifestation of the virus, especially with respect to virulence and transmission. SARS-CoV-1 (SARS1) spreads efficiently via the respiratory mode with greater virulence but not as efficiently as SARS-CoV-2 (SARS2). SDMs attribute these properties to higher N and M PIDs (N PID: 50%, M PID: 9%) in contrast to those of SARS-CoV-2. Figure 3B illustrates the mechanism of COVID-19 attenuation via pangolins, with an analogy from the Nipah virus (NiV) [4,18,32,33,82].

While Figure 3A summarizes the mechanisms in which infectiousness and virulence are modulated, Figure 3B shows the evolutionary implications of the mechanisms described. The greater virulence of SARS-CoV-1 is likely due to the brief period the virus spent in its intermediary host, the civet cat, before zoonotic transmission to humans. In contrast, the Nipah virus (NiV) is usually highly virulent when transmission to humans occurs directly from bats, as observed in the cases of Bangladesh and India. Meanwhile, the 1999–2000 outbreak involved the infection of humans via pigs as intermediary hosts, and the human CFR was approximately 50% compared to the 75% CFR in the cases from India and Bangladesh [4,33,36,82].

The finding, discovered via PONDR^®^-VLXT, that the disorder of inner shell proteins of the virus from the Malaysia outbreak was lower than those from the Bangladesh–India outbreak is consistent with the prediction of the virulence–inner SDM and disorder observations of viruses from the same family. The attenuation of the virus occurred during its infection of pigs, in which fecal–oral transmission is the most convenient route in a farm environment. The hardening of the inner shell protein poses several advantages. Firstly, the fecal–oral route does not necessitate higher disorder in its inner shell protein, and greater disorder requires greater energy, which is wasted if not utilized. Secondly, a harder inner shell offers extra protection for the viral genome. Bat CoVs, in general, have higher N disorder, possibly arising from the need for certain levels of respiratory transmission potentials because of the flight behavior of bats. Respiratory diseases, such as avian influenza, have been known to spread between birds during flight [83,84,85,86]. There is, therefore, no reason to doubt that respiratory viruses such as CoVs can spread between bats during flights. SDMs support this notion with respect to IBV, which is an avian coronavirus; it has the highest N PIDs, and the higher N PID is associated with higher respiratory transmission (Figure 2). Indeed, as we will see later, bat-CoV N PIDs are usually higher than those of most other CoVs.

Given the generally lower N PID and thus virulence, it is plausible that the precursor SARS-CoV-2 was deeply entrenched in an intermediary animal host. There are important reasons to believe that pangolins were involved. A smoking gun can be found in the abnormally hard M found in all COVID-19-related viruses, suggesting a burrowing animal. The presence of SARS-CoV-2-related pangolin-CoVs is apparently widespread in Southern China and Southeast Asia, as evidenced by the discoveries of pangolin-CoVs in Guangxi, Guangdong, and Vietnam [42,43,44,45,46,47,48,49]. Figure 3B highlights the pangolin as a likely reservoir, with pangolin-CoVs circulating among bats, humans, and pangolins.

### 3.5. Physiological Mechanisms Allow Dichotomy Between Virulence and Infectivity: Mucociliary Clearance (MCC)

It must be understood that the CoV-transmission SDM revolves around the need to have a sufficient concentration of infectious particles for the virus to have viable respiratory transmissibility. This can be facilitated by an abnormally hard M, increased disorder in N, or both, since a harder M makes the virion more resistant to damage arising from the antimicrobial enzymes found in mucus and saliva [21,22,23,24,25,26,27,28], whereas a more disordered N could induce more efficient viral replication as a result of more effective protein–protein binding. The latter is again used in the virulence–inner SDM, in which higher N disorder assists the rapid replication of viral particles, especially in vital organs. How do the two factors interact to allow us consistency among various SDMs? Part of the answer to this question is included in Figure 3A, which summarizes how virulence and infectiousness are intertwined. It does not, however, tell the whole story, especially regarding how the physiology of the host helps in the entire process.

**Figure 4:**
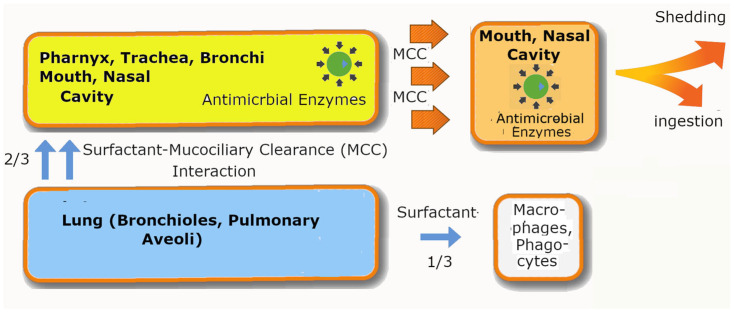
The mucociliary escalator/clearance (MCC) system. MCC consists of a network of mucus-covered structures found in ciliary cells. These mucus-covered structures push foreign materials away from the lungs towards the mouth and nasal cavity. MCC is absent in the lungs, where mucus is replaced by surfactants. About 2/3 of foreign matter in the lungs is expelled into MCC. The rest are engulfed by macrophages or remain in the lungs.

Knowledge of molecular physiology has allowed us to understand the roles that the respiratory system plays in the entire transmission process. Mucociliary clearance (MCC) or the mucociliary escalator is of particular interest [87,88,89,90]. It encompasses a body of knowledge that describes how the respiratory system expels foreign bodies, including viruses and bacteria, via a mechanism known as MCC. MCC is a sophisticated network of hairy ciliated cells covered with mucus that transport foreign particles away from the lungs towards the upper respiratory regions for removal, as shown in Figure 4. During the transportation process, the particle is exposed to antimicrobial enzymes in the mucus.

The three main types of cells in the respiratory tract are ciliated cells, goblet cells, and basal cells. We know that the ciliated cells are mucus-coated cells with hair-like structures used to move particles away from the lungs, while goblet cells produce the mucus [76]. In contrast, however, the lungs have different types of cells that do not contain mucus. It is likely that the antimicrobial enzymes in mucus and saliva are too harsh for the delicate function of the lungs. Instead, the mucus is replaced by surfactant. The cells found in the lungs are alveolar type I cells (AT1), alveolar type II (pneumocytes, AT2), and macrophages. AT1 secretes surfactants [90,91]. While surfactants are known to have some antimicrobial properties, they are incomparable to the myriad of antimicrobial enzymes found in the mucus.

Given these physiological mechanisms, we can now understand how the host body modulates the spread and virulence of SARS-CoV-1/2, as seen in Figure 3. When SARS-CoV-1 infects a patient, the greater N disorder allows for the rapid replication of a greater quantity of viral particles throughout the respiratory system. The particles are, however, subjected to MCC, in which the particles are swept upwards towards the nasal cavity, but by the time they reach the nostril, most infectious particles are eliminated or damaged due to the comparatively less rigid outer shell, M. The particles that are produced in the lungs tell a different story. Experiments have shown that the lungs partially participate in the MCC process by pushing 2/3 of foreign particles into the respiratory tract [92]. Since 1/3 of the original particles remain in the lungs, the remaining particles could pose a danger to the patient by damaging the lungs, especially when there are many of them. SARS-CoV-2, on the other hand, produces a more moderate amount of particles throughout the respiratory system, and many of the particles that are transported to the nostril via MCC remain undamaged, unlike in the case of SARS-CoV-2, as the former has an abnormally hard M.

### 3.6. Phylogenetic Trees Using M Reveal a More Intimate Relationship Between Pangolin-CoVs and SARS-CoV-2: Another Sign of a Pangolin Footprint

**Figure 5:**
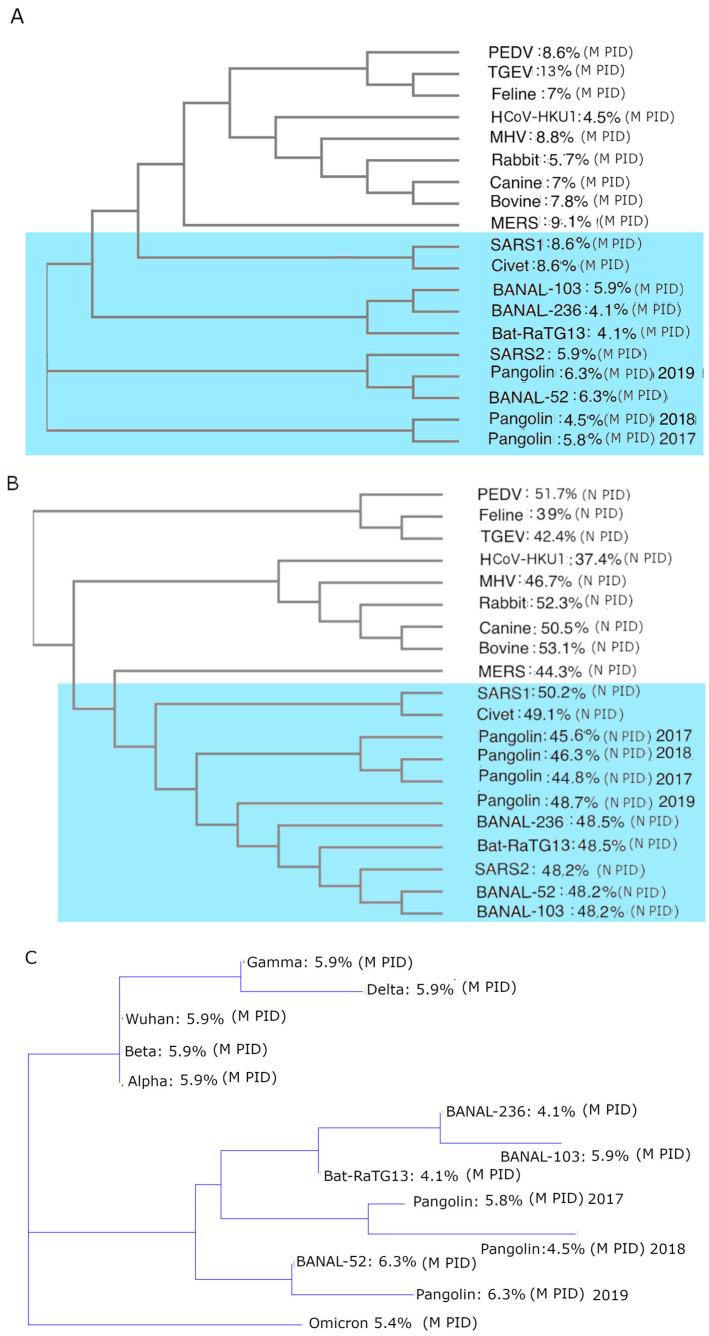
CoV phylogenetic study trees using M. (**A**) Phylogenetic study of CoVs using M via CLUSTAL OMEGA [88]. (**B**) Phylogenetic study of CoVs using N via CLUSTAL OMEGA [97,98]. (**C**) Phylogenetic study of COVID-19-related viruses using M (via CLUSTALW [99]). Viruses related to SARS-CoV-2/1 are shaded in blue in (**A**,**B**) [40].

We have seen that a hard M is the hallmark of all thus-far-known COVID-19 viruses. Being hard or ordered usually entails a more conserved protein. Based on this, M is very likely to be highly conserved among all COVID-19-related viruses. Such a feature renders M more ideal for phylogenetic studies, as it is known that recombinations could cause gross errors in phylogenetic calculations. Interestingly, phylogenetic calculations yielded results that are different from those using the entire genome or other proteins [41,42,43,44,45,46,47,48,52,55]. Figure 5A,C use M to show that pangolin-CoVs have a much closer relationship with SARS-CoV-2, which is not observed when other proteins such as N (Figure 5B) or the entire genome is used.

Figure 5A is different from Figure 5C as it involves slightly different algorithms. In any case, both show that pangolin-CoVs have intimate relationships with SARS-CoV-2. It is interesting to note that the two viruses that have the greatest sequence similarities to SAR-CoV-2 are bat-CoVs—specifically RaTG13 and BANAL-52 at 96.1% and 96.8%, respectively [51,52]. What is intriguing, however, is that in Figure 3A, BANAL-52 and Pang2019 have the closest relationship to SARS-CoV-2, in contrast to RaTG13. How can this be when RaTG13 has a 96.1% sequence identity to SARS-CoV-2 compared to about 90% for Pang2019? Sequence identity does not offer the full picture because of the possibility of recombination occurring. Furthermore, phylogenetic genetic algorithms tend to make mistakes when recombination has taken place [93]. The abnormally hard M found in all COVID-19-related viruses entails a structural and genetic conservation, which means that the likelihood of any recombination having taken place is much lower. Therefore, we believe that Figure 5A,C present the most accurate phylogenetic study by avoiding the chances of recombination.

Yet another odd feature can be observed in Figure 5C, where Omicron is more closely related to pangolin-CoVs than to the other variants [40,52,55]. While this may seem odd, we know that Omicron itself is shrouded in mystery. When Omicron was first sequenced, it was found to have mutations that are unlike any other variants. The question that quickly arises is this: Where has Omicron been hiding all along [94]? Why did the medical and scientific community not notice it if it was in the human population? There were a few suggestions. A few scientists suggested that the virus was hiding in a small group of immunocompromised people, such as HIV or cancer patients [95]. Others have suggested that the virus had been hiding in an animal such as a rat or pangolin. One study suggested that Omicron had been hiding among mice based on the mutations of its S protein and mouse ACE-2 [95]. Disorder studies on Omicron suggest that it could be hiding in a burrowing animal, such as a pangolin, as the first Omicron variant BA1 had an even harder M than other SARS-CoV-2 variants (M PIDs: 5.4% vs. 5.8%) [55]. The idea that Omicron had been hiding in mice actually does not contradict the suggestion in the previous statement, since mice are also burrowing animals. A complication arises, however, when we examine the evolution of mice and rats. While rats and mice dwell in burrows in the countryside, rats and mice in urban settings have evolved to live in human homes (Schmidt-Holmes et al.) [96]. Therefore, depending on the species, they could have characteristics of both burrowing and non-burrowing animals. The phylogenetic tree (Figure 5C) that shows an unusually close relationship between Omicron and pangolin-CoVs, in contrast to the other variants, adds an important clue towards solving the puzzle. The phylogenetic tree suggests that it was literally hiding within a population of a burrowing animal, such as pangolins.

## 4. The Shell Disorder Models and Reproducibility: M and N Proteins

### 4.1. The Problem with the S Protein: Limitations and Potentials

When COVID-19 first struck in Wuhan, it spread globally with a ferocity not seen since SARS-CoV-1, despite efforts to control it. Almost immediately, many scientists began to zero in on S as the protein responsible for its high infectivity [9,10,11]. A computational model suggested that SARS-CoV-2 S binds more efficiently to the ACE-2 receptor than SARS-CoV-1 [9,10,11]. Furthermore, a specific polybasic sequence known as the furin cleavage site (FCS) can only be found in SARS-CoV-2 but not in the COVID-19-related bat-CoVs, pangolin-CoV, and SARS-CoV-1 [100,101,102]. Many scientists hailed S, as well as FCS, as the holy grail of COVID-19 transmissibility and virulence knowledge [101,102].

The S protein has been widely studied [103] since it plays important roles with many implications. It is intimately involved in viral entry, which presents an opportunity for the discovery of drugs and vaccines that block the attachment of the virus to the host cells [103]. Furthermore, being a surface protein, it is widely studied for the way its mutations help evade the host immune system. It is therefore not difficult to see why S is the most studied CoV protein. S is, however, so intensively studied that one might get the impression it is the only protein that matters or that no other CoV proteins exist. This paradigm is, of course, nonsensical as it violates a basic tenet of biochemistry: each protein plays individual but important roles [104].

Even in the debate involving COVID-19’s origin, many scientists seem to imply that the secret of COVID-19 infectiousness and virulence lies in the S protein and that the moment the secret of S is unlocked, the origin of COVID-19 will be uncovered as well [101,102]. Contrary to the popular notion, there is mounting clinical and experimental evidence that the S protein may not be the main underlying cause of COVID-19 infectiousness and virulence. One major piece of evidence for this lies in a comprehensive clinical study conducted by Wolfel et al. [29]. In this study, it was found that COVID-19 patients shed substantially more infectious particles than SARS-CoV-1 patients. If we look closely, the question that comes to mind is as follows: Why does SARS-CoV-2 require substantially more particles to be more infectious if its S has a 10 or 1000 times [9,10,11] greater binding affinity to ACE-2 than the latter? It is important to keep in mind that producing additional infectious particles requires substantial energy, and nature does not generally expend energy on redundant processes. One could, however, attempt to navigate this paradox by claiming that the S affinity to ACE-2 is responsible for the more rapid replication of the virus. Unfortunately, this suggestion contradicts what we know about the life cycle of the virus, as viral entry represents only the initial stage in a long series of events that includes RNA replication, protein production, assembly, packaging, and the budding of viral particles [105,106,107,108].

There are those who suggest that it is possible that SARS-CoV-1 S binds more efficiently to ACE-2 in cells in the lower respiratory tract in contrast to SARS-CoV-2 [12]. There are a number of issues that arise when such an argument is made. Firstly, the argument is made without considering current physiological knowledge, namely, the mucociliary clearance system (MCC), which allows viral particles produced throughout the respiratory system to be transported upwards towards the nasal area such that they can be expelled and shed [87,88,89,90]. Secondly, to our knowledge, attempts to reproduce the argument involving SARS-CoV-1 S and ACE-2 have not been conducted experimentally, probably because of the current difficulty in obtaining the now extinct SARS-CoV-1 to conduct comparative experiments alongside SARS-CoV-2. There are, however, also similar arguments made to account for the differences in virulence of Omicron and non-Omicron variants (Hui et al. [74]). The difference is that the COVID-19 pandemic provides us with a deluge of clinical and experimental data. In fact, attempts to reproduce this argument have been carried out, which we will discuss at length later.

The clinical study of Wolfel et al. [29] is only the tip of the iceberg, as there is also a deluge of other evidence that shows that the nature of S is not what many scientists have made it out to be. Nor does S alone provide for a coherent conceptual framework of COVID-19 infectiousness and virulence, unlike M and N. This is also the case with long COVID. We will examine these in greater detail in later sections. This is not to say that S is unimportant or that it does not play any part in infectiousness or virulence. It is important, but we must also fully comprehend its true potential and limitations. A more complete understanding of the true limitations and potentials of S will come when we study other important proteins, such as N and M, more thoroughly, which is challenging even if the majority of research is oriented towards S.

### 4.2. SDMs and Reproducibility

We have seen that SDMs are highly reproducible due to their ability to accurately predict and explain certain phenomena that are otherwise difficult to account for. Unlike alternative explanations, they are explained in a coherent manner using a logical and unified paradigm that is consistent with current physiology and biochemistry knowledge [52]. There is also important clinical and experimental evidence that reproduces many of the predictions of SDMs.

While a previous experiment was not able to detect any statistical difference in the ability of SARS-CoV-2 to last on various surfaces in the presence of light when compared to SARS-CoV-1, Riddell et al. [21] conducted a similar experiment devoid of light, and they found that SARS-CoV-2 lasts substantially longer on external surfaces than the control CoVs. SDMs predict that SARS-CoV-2 is more persistent than most viruses as its unusually hard M protects against the environment and harsh antimicrobial enzymes, and this experiment reaffirms SARS-CoV-2’s resilience in the absence of light.

The reproducibility of SDMs is not only confined to computational and experimental research but also extends to clinical studies. One such study involved an investigation into the infectiousness of SARS-CoV-19. It was found that COVID-19 patients shed substantially larger amounts of infectious particles than 2003-SARS patients [29]. This contradicts all other paradigms set forth. For instance, if S is fully responsible and has a much greater affinity for human ACE-2, why does SARS-CoV-2 need to expunge such higher quantities of particles in order to be more infectious? SDMs provide for a substantially more elegant explanation: The virus is more resistant to the salivary and mucosal antimicrobial enzymes because of the abnormally hard SARS-CoV-2 outer shell (low M PID). This clinical observation raises more questions than it answers. More specifically, how is the virus able to induce the host to expel such a large number of particles without being more virulent to the body? If we assume that the body is shedding more viral particles because it is producing more virus copies, then vital organs such as the lungs should be flooded with the virus, thus making the latter more dangerous, but this is apparently not happening in the case of COVID-19.

Adding to this paradox, Ogando et al. [75] found that SARS-CoV-1 exhibits higher viral growth in VERO-E6 cells than SARS-CoV-2 under the same conditions. Even if this is consistent with the greater pathogenesis of SARS-CoV-2, how do we then reconcile this result with the previously mentioned clinical observation? If SARS-CoV-2 S has a 10 or 1000 times [9,10,11] greater affinity for ACE-2 than the affinity between SARS-CoV-1 S and ACE-2, how is this possible? It seems that we need to look elsewhere for answers by examining N and M more closely. The SDMs explain that the reason SARS-CoV-1 is producing higher levels of particles is related to the higher N disorder that allows greater efficiencies in its replication process; however, conversely, fewer infectious particles are shed by the body as the higher M PID does not provide sufficient protection against antimicrobial enzymes.

Yet another enigma involves long COVID [109,110], and SDMs can explain the persistence of the virus among COVID-19 patients even months after infection. Again, we return to the theme of hard M that protects the virus from antimicrobial enzymes. This time, the focus is not only on mucosal and salivary enzymes as in the case of infectivity but also on antimicrobial enzymes in the immune system. Further discussion on the antimicrobial enzymes found in the immune system can be found in the long COVID section below.

### 4.3. More Reproducibility: Omicrons and Pangolin-CoVs

There are many other predictions that SDMs make involving N and M. One such prediction pertains to pangolin-CoV. When SDMs were applied to pangolin-CoVs, it became obvious that the 2017 pangolin-CoV (Pang2017) isolate from Guangxi is attenuated because of its low N PID (~44%) [40,52,55,56]. There are several implications for this finding. If this or a similar virus had entered the human population, it likely would have spread quietly among humans as a mild cold, without attracting the attention of the medical community [18]. The predicted attenuation has been independently reproduced by several laboratories [52,55,70,71,72,73]. Animal models have observed milder manifestations of symptoms upon Pang2017 infection, in contrast to the Wuhan-Hu-1 strain. The SDM prediction pertaining to Pang2017 was published before the arrival of Omicron.

Omicron was first detected in South Africa around November 2021. Omicron was clinically and, later, experimentally observed to be milder than previous variants [70,71,72,73,74,79,80,81]. Once again, SDMs offer important insights. Based on the initial Omicron BA.1 subvariant, the N and M PIDs are 43.65% and 5.4%, which are both lower than previous variants [49]. The smaller-than-usual M PID (5.9% vs. 5.4%) could suggest that the virus had a recent origin involving a burrowing animal, whereas the lower N PID predicts that Omicron is likely attenuated to similar levels as Pang2017. The prediction involving N PIDs was experimentally replicated when it was shown that the viral growth of VERO-E6 cells infected by Pang2017 and Omicron is very similar and when it was shown that the viral damage to cells by the two viruses is similar [55]. This presents evidence of further reproducibility for both Pang2017 and Omicron. Omicron has also been shown to be attenuated with lower growth than Wuhan-Hu-1 under viral titration [55,71,73,75].

The correlations between N disorder and viral titer or virulence can be found in SARS-CoV-2-related virus data that include Pang2019 [71,79,80,81] and the Laotian bats-CoV (BANAL) [50,51]. Furthermore, while Pang2017 has been predicted and reaffirmed to be attenuated, this is not the case with Pang2019. Instead, SDMs have to predict that Pang2019 is non-attenuated with its N PID at 48.2% [18,52,55,56]. Huo et al. [77] were able to obtain levels of viral titer from Pang2019 similar to those of Wuhan-Hu-1 in VERO-E6 cells. Several laboratories were also able to independently observe that Pang2019 can inflict severe disease in at least one strain of mice. In contrast, virulence was not observed in Pang2017 [55,70,71,72,73,74,75]. All these are again consistent with the predictions of SDMs. We know that all of these SARS-CoV-2-related viruses do not possess FCS, unlike SARS-CoV-2. The question then becomes the following: how does Pang2019 possess similar infectivity and virulence as SARS-CoV-2 without FCS when FCS-mutant non-Omicron SARS-CoV-2 is largely not transmissible among ferrets?

### 4.4. Measuring Virulence Using CFR, Animal Models, and Cell/Tissue Damage Observation

While Figure 1 shows a correlation between inner shell disorder and CFR, it must be admitted that CFR is not necessarily the most ideal representation of virulence for at least two reasons. Firstly, CFR figures are often extrapolated out of necessity [102]. Secondly, CFR is applied only to humans, not animals. Furthermore, virulence may vary among different animals even within a single virus or variant. There are, however, other methods of measuring virulence, such as animal models, viral titration, and indications of cell/tissue damage after infection. We have mentioned some of the animal models. It must be noted that the attenuation and aggressiveness of SARS-CoV-2 variants, Pang2017-CoV and Pang2019-CoV, were reproduced by viral titrations, animal models, and inspections of cell/tissue damage after infecting cells or animals, and these experiments were conducted by at least two independent laboratories for each virus or variant [55,70,71,72,73,74,75]. The animal models included, at least, hamsters and several strains of mice. In addition, the severity of Pang2019-CoV infection was also observed in pangolins under laboratory conditions [76,77,78]. It can also be argued that a SARS-CoV-2-related virus may infect different types of cells in different ways, which could potentially render the interpretation of viral titration data more challenging. This is related to the argument made by some scientists that SARS-CoV-1 could infect the lower respiratory tract more efficiently in comparison to SARS-CoV-2. There are, however, hints that the arguments may not present great difficulties in the interpretation of viral titration data. Viral titrations have been created with respect to a variety of cells, including Vero E6, Calu3, and Caco2 [73]. The data show subtle but definite differences in the viral titers across three different cell lines, resulting from variations in the S-ACE-2 binding mechanism. The differences, however, are not so substantial that they would render the interpretation of viral titration data challenging, as the viral titer variations among cell types are not substantial and follow a trend. In addition, as observed later, the S of SARS-CoV-2-related viruses typically becomes more efficient in binding with the S of different cell types after several passages. With all this in mind, it is possible to present a consistent picture of virulence and infectivity. In fact, a previous study [55] was able to find a positive correlation between virulence and N PIDs based on a combination of data from viral titers, animal models, and cell plaques.

## 5. The Roles of S, M, and N in Viral Replication: An Enigma

### 5.1. The S Protein, Omicron, and Pangolins

We have seen that the use of S is unable to explain some crucial experimental and clinical data. This is only the tip of the iceberg, as there are more experimental and clinical data that S alone simply cannot explain or account for; moreover, thus far, attempts to do so are not reproducible or can be shown to be inherently flawed, as we have argued. There are also important biological reasons for this. For us to understand this phenomenon, we need to examine the basic fundamental biology of the virus. While S is definitely an important protein in terms of viral entry, there is no fundamental reason that S should be the most important protein in COVID-19 infectiousness and virulence, as many scientists would like to believe. Viral entry, though important, is only the first of many steps in the virus’s life cycle [108]. It must be emphasized that SARS-CoV-2 encodes 19 proteins, even though not all are major proteins [108]. S is not the only major protein, and neither is it the most abundant protein. On the contrary, N and M are the most abundant major structural proteins found in the cell and virion, respectively [95,96,97,98,99]. For this reason alone, we should not be surprised if N and M have greater influence on the infectivity and pathogenesis of the virus.

Further sets of perplexing evidence that contradict the “S-alone” paradigm can be found in data that pertain to pangolin-CoVs and Omicron. We need to keep in mind that all variants of SARS-CoV-2 have FCS, which is absent in all COVID-19-related bat and pangolin viruses. Many scientists thought that they had found FCS to be the true cause of COVID-19 infectiousness and virulence when, instead, it was found that SARS-CoV-2 with the FCS intact is more aggressive in respiratory cells—in contrast to the FCS-mutated one—by promoting cell-to-cell fusion. Furthermore, it has been shown that while the non-Omicron SARS-CoV-2 wild-type can be transmitted experimentally via aerosol between hamsters, no transmission was observed when the FCS-mutant was used on ferrets [111]. Again, how can this be the case, given that pangolin-CoV has no FCS and is easily transmitted between hamsters [70,71,77]?

### 5.2. The S and Omicron Conundrum

When Omicron emerged, it presented an enigma by being infectious yet attenuated. How did Omicron achieve its attenuation given that it has an FCS similarly to all other variants? Animal studies have shown that rats are unable to transmit the virus via aerosols in the laboratory. How could this be so when Omicron has been clinically shown to be highly infectious? More importantly, how can this occur when Omicron has an efficient FCS [112,113], in which its presence in other variants has been shown to induce greater transmissibility in ferrets [111]? These discrepancies point to the probability that other factors are in play. In fact, all these, as we have shown, are consistent with the workings of N and M, as summarized in Figure 5.

An attempt to navigate this “S-Omicron” paradox is the hypothesis that claims Omicron infects the cells in the upper respiratory system more easily than the lungs. Hui et al. [74] were able to isolate bronchial and lung tissues, which were infected with Omicron and previous COVID-19 variants. They were able to qualitatively observe the greater presence of viral particles and concluded that Omicron is attenuated because it replicates more easily in the lungs than in the bronchi. There are, however, several problems with this interpretation. Firstly, this observation has not, to our knowledge, been reproduced in other laboratories. In fact, several laboratories have observed efficient replications in both lungs and upper respiratory systems [73,113,114]. Furthermore, several other independent laboratories have observed that many subvariants of Omicron are even more adapted to the human ACE-2 than the non-Omicron variants [113,114,115]. If this is the case, why has Omicron not achieved greater virulence and infectivity similar to its predecessors?

### 5.3. Evidence of the Different Roles of N and M in Experimental Data

The story is actually even more complicated than what Hui et al. had envisaged [74]. In reality, the N and M proteins, together with knowledge of MCC, provide a more consistent and reproducible explanation for their results. In our previous publications [40,52,55], we have shown that the initial waves of Omicron had lower M PIDs than previous variants. This feature is likely a tell-tale sign of its recent interactions with a burrowing animal, possibly the pangolin. In any case, it explains why Hui et al. were able to observe more viral particles in the bronchi. It is because Omicron is more resistant to the antimicrobial mucosal enzymes that it encounters as it is being transported upwards by hair-like structures in the network of mucus-covered ciliary cells. The lungs lack ciliary cells or mucus but contain surfactants that, although antimicrobial, are less harsh than the diverse array of enzymes present. The apparent lack of viral particles is likely an indication of particles still trapped in the tissues, as there would not be MCC to bring them to the top of the lungs [87,88,89,90,91].

**Figure 6:**
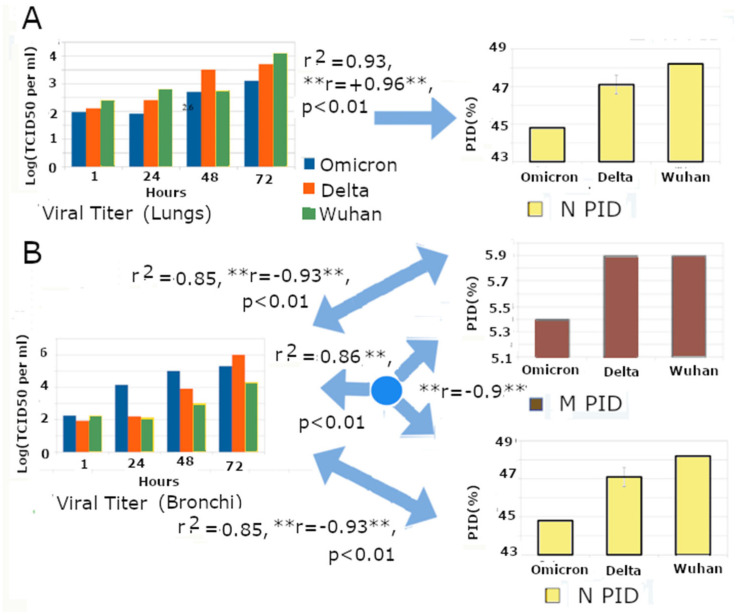
Multivariate analysis of M/N disorder and viral titer [40]. (**A**) Regression analysis reveals a positive correlation between N PID and the viral titer from human lung tissues and N PID (model: VT = A * (N PID) + B * Time + C, where VT = viral titer, A, B = coefficients, and C = y-intercept). (**B**) Regression analysis highlighted a negative correlation between viral titers from human bronchial tissues and M/N PIDs (regression model: VT = A * (M PID) + B (NPID) + C * Time + D, where VT = viral titer, A, B, C = coefficients, and D = y-intercept). The analysis is a statistical extension of the experiment of Hui et al. [40,43]. Data pertaining to viral titrations and PIDs are from the experiment of Hui et al. and available in Table 2 (which can also be found in previous publications). Note: The correlation signs seen in r (**) for (**A**,**B**) are different.

Viral titrations were also carried out using Omicron, Delta, and Wuhan-Hu-1 in lungs and bronchial tissues in the above-mentioned experiment. The data were used to support the hypothesis of the differentiated replication of Omicron in the lungs and bronchi. In one of our previous articles [55], we managed to use their data to perform multivariate analysis (Figure 6). The viral titration data were obtained from a publicly available manuscript published by Hui et al., while N and M PIDs—based on variants and sub-variants—were obtained from our curated disorder database (Table 2). Regression (multivariate) analysis was performed using the R package. The regression analysis provides us with correlations between viral titers, and N/M PID is measured as correlation coefficients (r) or coefficients of determination (r^2^). The total n (sample size) for the entire statistical experiment, as shown in Figure 6, is 24 (*p* < 00,1, n = 24, r^2^ ~0.9). Statistical results were computed using the R package, which is available publicly [116,117].

**Figure 7:**
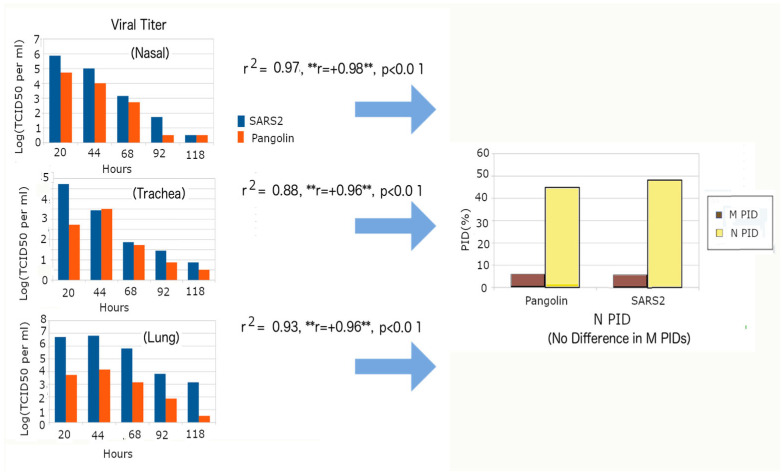
Multivariate analysis of N/M disorder (PID) and viral titer of pangolin-CoV/SARS-CoV-2 in VERO-E6 (regression: VT = A * (N PID) + B * Time + C, where VT = viral titer, A, B = coefficients, and C = intercept) [40]. The results are a statistical extension of the experiment of Guo et al. [40,71]. Viral titration data are from the experiment of Guo et al., whereas PID data are available in Table 2 and previous publications. Note: The correlation signs seen in r (**) are both positive unlike Figure 6.

We found strong correlations between N/M PIDs and viral titers. We were also able to observe peculiar changes in the signs of the correlations when moved from lung tissues to bronchial ones [40]. We were able to obtain a positive correlation (r = +0.96, Figure 6A), especially with respect to the N PID and viral titer (VT = A * PID_N_ + B * Time + C, where A, B, C = coefficients and VT = viral titer). It was, however, very startling when we received a negative correlation (r = −0.93, Figure 6B) between PID_M_/PID_N_ and the viral titer (VT = A * PID_M_ + B * PID_N_ + C * Time + D, where VT = viral titer, A, B, C = coefficients, and D = Y-intercept) [44]. What is remarkable and puzzling is the change between the positive and negative signs. It is not only reproducing the SDMs but also instructing us on how to use the SDMs. The change in the sign is an indication that the greater presence of particles in the lungs is dependent on the greater N disorder (higher PIDN), whereas the greater viral presence in the bronchi is dependent on lower disorder in N and M PID (lower M PID and N PID). We will also see that this change in slope (correlation) is completely absent when viral titration is conducted in an animal model instead of tissues (Figure 7), as viral particles can easily travel upward via MCC in the case of animal models.

Prior to this investigation, we focused on the hard outer shell (low M PID) with respect to its resistance to the onslaught of mucosal antimicrobial enzymes and on greater N disorder with respect to its ability to assist in providing more efficient viral replication [18,19,20], but the experiment also showed us that N plays a role in protecting the virion from damage [52,55]. This is something we had overlooked, even though evidence from the very beginning indicated that harder inner shells protect viruses such as NiV, EIAV, DENV, and rabies [34,35,55,56,65,66,67].

We conducted a similar regression study, as observed in Figure 7, using the experimental data of Guo et al. [71]. Again, we were able to obtain titration data from the published study of Guo et al. with the respective N and M PIDs, which depend on the SARS-CoV-2 variant and pangolin-CoV isolate, obtained from our curated database. As with the data from Hui et al., we carried out regression analysis to obtain the correlation coefficients (r) and coefficients of determination (r^2^), which were grouped according to the samples’ location in the respiratory system (Figure 7, total n = 30, *p* < 0.01, r^2^ ~0.9).

Guo et al. used Pang2017 and hamsters instead of Omicron and tissue cultures, respectively [65]. Therefore, M becomes an unreliable independent variable as there is hardly any difference between the M PID of Pang2017 and non-Omicron SARS-CoV-2. We also expected to observe the full effect of MCC since Guo et al. [71] used an animal model, in contrast to the use of tissues by Hui et al. [74], i.e., the viral particles are more able to move freely between different parts of the respiratory system via MCC. Positive correlations between N PID and viral titer were observed in samples collected from all three areas of the respiratory system, as observed in Figure 7, which helps validate SDMs and the results described in the previous sections.

### 5.4. Hui et al. Experiment: S-Alone Hypothesis vs. SDMs

The S-alone hypothesis argues that SARS-CoV-1 infects the lower respiratory tract more easily as its S binds more efficiently to the cells in the lower respiratory system. The same argument has been made to account for the lower virulence of Omicron compared to other variants. Hui et al. tried to show this phenomenon. They cultured various variants in bronchial and lung tissues. Their viral titration data show lower viral titers for Omicron in the lungs than in the bronchial tissues. When we applied their viral titration data to carry out more elaborate statistical analysis correlating viral titers with N and M PIDs, our results, however, revealed a different pattern [40]. There are strong correlations between viral titers and N/M PIDs in both bronchial and lung tissues. Moreover, positive and negative correlations were found in the lung and tissues, respectively. The results not only reproduce the predictions of SDMs but also provide insight into how SDMs should be interpreted. The positive correlation between N PIDs and viral titers in the lungs reveals that the greater presence of viral particles is due to the greater N disorder (PID), which depends on the variant—keeping in mind that the lungs have surfactants in lieu of mucus. Surfactants exert some antimicrobial effects, but these are far less potent than the diverse array of antimicrobial enzymes present in mucus. This is the reason why there is a strong positive correlation between viral titers in the lung tissues and N PID and a weaker correlation with M PID. The puzzling question is then the following: Why is there a negative correlation between viral titers in bronchial tissues and N/M PIDs? The answer is related to the fact that, in contrast to the lungs, bronchial tissues, similarly to most upper respiratory tissues, have mucus. A negatively correlated M PID means that the hard M is protecting the virus from antimicrobial damage. What we did not expect was a correlation with N disorder. At that point, we realized that this result indicates that N also plays a role in protecting the virion from damage, especially when exposed to an onslaught of antimicrobial enzymes. The results reveal how predictions can be interpreted using SDMs. Indeed, we had previously observed that some viruses, such as the rabies virus (RABV), with harder inner and outer shells thrive better in harsh environments, such as exposure to saliva [65,66,67]. It must also be noted that M and N bind to each other in close proximity in the virion. Further literature searches have provided experimental evidence that M and N, taken together, permit greater structural integrity via the non-covalent binding between them. This provides support for computational results that suggest that both M and N protect the virion from damage.

Our more elaborate statistical analysis indicates that the higher viral titer of Omicron is not due to an increased number of viral particles in the bronchial tissues, nor due to differences in the S protein. Rather, it arises from the virus’s enhanced ability to resist damage from the abundant mucosal antimicrobial enzymes present in the bronchial tissues. Omicron’s greater resistance to antimicrobial enzymes arises from its greater hardness in both M and N (N PID: 44.8%; M PID: 5.4%). The particular Omicron variant used was BA1, which was the original subvariant that was first detected in South Africa. While all Omicron variants have lower N PIDs, BA1 has the peculiar characteristic of an even lower M PID (~5.4%) than all other variants or Omicron subvariants (~5.9%) [55]. As a result of this phenomenon and its harder N, it can resist antimicrobial enzymes in the bronchial tissues; thus, there is a greater presence of viral particles. In the lung tissues, however, there is a lesser presence of antimicrobial enzymes; however, because Omicron’s N PID is substantially lower, it is unable to replicate as fast as the other variants, especially in the lungs. This phenomenon is detected by the more elaborate statistics applied, and this result is arguably more plausible than the one presented by Hui et al. [74], as the former shows a more intricate reason for the results of the latter.

### 5.5. The Role of MCC in Virulence and Infectivity

Puzzling and seemingly contradictory results are observed in the viral titration of pangolins, as seen in Figure 7 (Guo et al. [71]). Unlike the statistical findings from Hui et al.’s data, the statistical analysis using the data from Guo et al. [71] shows positive correlations for viral titers with respect to N PIDs in both the upper and lower respiratory tracts. While this may appear to contradict the results we just discussed, it is not actually inconsistent. The experiment conducted by Guo et al. was based on live hamsters infected with pangolin-CoV. They measured the viral titers by extracting samples from euthanized hamsters. Hui et al., on the other hand, relied solely on viruses grown in bronchial and lung tissues. Therefore, we should expect the full effects of MCC in the experiment of Guo et al., but only limited MCC effects are observed in the experiment of Hui et al. because the viral particles produced across the respiratory system—including the lower respiratory system—will move upward towards the nasal region for expulsion. This is what the statistical results shown in Figure 7, unlike those in Figure 6, are revealing. The results of Figure 6 and Figure 7 cast doubt on the hypothesis that SARS-CoV-1 is less infectious but more virulent due to its S protein binding more efficiently to ACE-2 cells in the lower respiratory tract. This is because MCC transports much of the viral particles produced in the lower respiratory tract upward to the nasal region for expulsion from the body. The same argument has been made for the more virulent SARS-CoV-2 variants. It seems that a more plausible scenario is that the virus replicates approximately the same amount throughout the respiratory system. The more virulent strain or variant with higher N PID replicates extensively throughout the respiratory system, particularly in the lungs. However, the extent to which viral particles are expelled from the body depends on the virus’s ability to withstand the onslaught of mucosal and salivary antimicrobial enzymes via a harder M.

### 5.6. SDMs Account for MCC in Virulence and Infectivity

One problem with the “S-Alone” hypothesis involving SARS-CoV-1/2 virulence and infectivity is that it is oblivious to current physiological knowledge, namely, MCC. Even if SARS-CoV-1 does bind more efficiently to the lower respiratory tract compared to the upper respiratory tract, thereby rendering SARS-CoV-1 less infectious but more virulent, MCC entails that the viral particles will move upward towards the nasal region to be expelled. This, of course, assumes that the viral particles will survive the onslaught of antimicrobial enzymes during transportation by mucus-covered ciliary cells. The MCC factor must be considered if we attempt to demonstrate the difference in virulence and infectivity between Omicron and non-Omicron variants. Because MCC is an important principle in physiology, MCC must therefore be essential in explaining the mechanisms of virulence and infectivity manifestations. SDMs, unlike the S-alone hypothesis, can account for MCC, and MCC can explain the link between infectivity and virulence via SDMs. MCC and SDMs, therefore, complement each other in accounting for differences in the infectivity and virulence manifestations of SARS-C0V-1/2 and SARS-CoV-2 variants.

### 5.7. The HCoV-NL63 Enigma

The above sections do not imply that S plays no role in infectivity and virulence; rather, they indicate that S does not contribute to infectivity and virulence in many of the ways commonly assumed. Its roles should be evaluated in the broader context, alongside other proteins such as N and M. To understand it further, we turn our attention to another human CoV. The only other known human CoV (HCoV)—other than SARS-CoV-1/2—that binds to ACE-2 is NL63 [118,119,120,121,122,123]. HCoV-NL30 usually manifests itself as a mild cold even though it known to be capable of infecting both the lower and upper respiratory tracts. NL63 and SARS-CoV-1/2 are not closely related, as they comprise alphacoronvirus and betacoronavirus, respectively [123]. It can be argued that NL63 is somewhat less infectious than SARS-CoV-2 since the former usually only infects children and immunocompromised individuals, such as the elderly, unlike the latter.

NL63 has no FCS recognition site even if an FCS sequence has been detected at S2 [120,121]. While cleavage is seen at the Golgi apparatus of cells infected by SARS-CoV-2, it is not observed in NL63 infection. Keeping in mind that SARS-CoV-1 does not have FCS, unlike SARS-CoV-2 [100,101,102], the absence of an FCS recognition site presents an intriguing enigma. If NL63 and SARS-CoV1 have no FCS recognition site, why is NL63 obviously less infectious than SARS-CoV-1? Conversely, if SARS-CoV-2 has an FCS recognition site, unlike NL63 and SARS-CoV-1, why does SARS-CoV-2 exhibit infectivity that is comparable to NL63 when compared to SARS-CoV-1, given the fact that both COVID-19 and NL63, but not SARS-CoV-1, are endemic? We know that SARS-CoV-1 has limited infectivity as there were only 8422 known cases of infection, and it is now extinct, whereas COVID-19 and NL63 are endemic even to this day [3,12,123]. All these phenomena seem to contradict the idea that the presence of FCS is responsible for higher infectivity [101,102]. As for pathogenesis, why are SARS-CoV-2 and NL63 substantially less virulent than SARS-CoV-1 when SARS-CoV-2 is the only virus of the three that has an FCS recognition site given that the presence of FCS is associated with greater pathogenesis?

Likewise, the relationship between S-ACE-2 affinity and pathogenesis or infectivity can cause substantial confusion, especially when NL63 is taken into consideration. We have observed that the affinity of SARS-CoV-2 S with respect to human ACE-2 is much higher than that of SARS-CoV-1. Many scientists have postulated that this discrepancy is responsible for SARS-CoV-2’s higher infectivity, especially among humans [9,10,11]. This postulation is, however, unreproducible, as it has been shown that NL63 S’s affinity for ACE-2 is less than that of SARS-CoV-1 [123]. How could this be possible when NL63 is obviously substantially more infectious than SARS-CoV-1 for the reasons already mentioned? All these questions form a conundrum that is difficult to explain by S alone and is more effectively explained when the different roles of S, N, and M are taken into consideration.

This virus is, however, also enigmatic for SDMs, as its N disorder is very close to that of SARS-CoV-1 (N PIDs: 49.8% vs. 50.2%), but NL63 is, of course, not as virulent. Does this mean that SDMs are wrong? If we look closely, we will find that this is not necessarily true. SDMs usually function better when the compared viruses are closely related. NL63 is not closely related to SARS-CoV-1/2; as a result, the proteins, in general, are not similar between the two. Because of the differences in proteins, especially those beyond shell proteins, other factors can come into play, such as differences in the toxicities of other proteins. This is a limitation of SDMs.

Nevertheless, in this case, comparisons using SDMs are possible in this case. While the NL63 N PID is higher than SARS-CoV-2 but comparable to SARS-CoV-1, the M disorder tells a different story. It is substantially higher than that of SARS-CoV-1/2 (NL63 M PID: ~11%; SARS-CoV-2 M PID: 5.8–54.%; SARS-CoV-1 M PID: ~9%). Applying current knowledge from SDMs, the relatively soft NL63 M and N proteins imply that, although a comparatively large number of viral particles may be produced even in the lower respiratory tract, these particles are easily damaged by mucosal antimicrobial enzymes as soon as they are produced. It is for this reason that NL63 is usually not dangerous. If we scrutinize the clinical data more carefully, however, we observe that NL63 is capable of infecting both the upper and lower respiratory tracts [118,119] and that the individuals it infects in this manner are mainly children and immunocompromised adults, such as the elderly. These clinical manifestations may be the result of its ability to replicate in larger quantities in all areas of the respiratory system if unchecked by the immune system, including the antimicrobial enzymes in the mucus. SDMs also suggest that, despite its greater vulnerability to damage by mucosal antimicrobial enzymes, some particles can reach the nasal region to be shed because greater quantities are produced as a result of a more disordered N. The fact that children and immunocompromised adults are more easily infected could provide further support to the idea that only limited quantities of viral particles are shed. Children often play in close contact with each other, especially at preschools.

### 5.8. The Protective Roles of Outer and Inner Shells

We have observed how both the outer (M) and inner (N) shells play roles in protecting SARS-CoV-2 from antimicrobial enzymes in the mucus and saliva. Apparently, this is not the only virus to exhibit such properties. There are several studies that report that viruses exposed to saliva usually have hard outer shells and often also have hard inner shells. Examples of such viruses are the EIAV, rabies virus, and Zika virus [4,34,35,66,67]. EIAV is transmitted between horses via horseflies that suck the blood of an infected horse, storing it in their mouthpiece with their saliva before sucking the blood of a new host, which is thereby infected. The rabies virus resides near the salivary gland of the host and is therefore exposed to saliva. Similarly, ZIKV is exposed to saliva when it is retained in the mouth of an Aedes mosquito during feeding on the blood of an infected host. These phenomena, however, do not necessarily indicate that all viruses have a degree of inner shell hardness, as all viruses exposed to saliva usually have hard outer shells. This is the case in the flavivirus family, in which the viruses are usually insect-borne and have a hard outer M, but a few viruses, such as ZIKV, also have a harder capsid.

### 5.9. The Functions of M and N

A great way to gain more insights into the structure, nature, and functions of viral proteins, such as N and M, is by inspecting SARS-CoV-2 using microscopy, particularly electron microscopy (EM) [124,125,126,127]. Observations of the SARS-CoV-2 virion, including M and N, did not reveal anything unusual that is not seen in other CoVs. M spans the viral membrane, which is a lipid bilayer of about 5 nm, with the former protruding approximately 2.5 nm, which is not unusual for CoVs [124,125,126,128]. Inspections of the SARS-CoV-2 using powerful EM have revealed the reasons that M and N are the most abundant proteins in the virion and cell, respectively. M spans the entire membrane, which encloses the entire virion, whereas N encases the RNA genome [124,125]. The structural arrangement itself suggests protective roles of the two proteins. M binds strongly to both S and N. The affinities for S and N allow M and N to play important roles in the assembly and budding of viral particles [106]. Because of the strong affinity between M and N, the greater flexibility in N provides for more efficient recognition between N and M [16,30,31,32]. This is one of the reasons that greater N disorder allows faster and more effective viral replication.

There is yet another property of M and N that has been observed using EM. This involves the nature of M and N binding. Powerful EM techniques were used to observe that the C-terminus of M binds to N via an ionic interaction [129]; moreover, in the absence of this interaction, the entire virion collapses under harsh conditions, such as high salt or PH levels. This important observation provides support to the suggestion that M and N play roles in protecting the virion. While M protects the entire virion, N is likely to specifically protect the genomic RNA, as N encases the RNA; thus, strong affinities between the two entities exist [105,106,107,108]. N’s affinity to the viral RNA has provided N with many important roles, especially those pertaining to viral replication. This includes assembly and RNA transcription [106,107,108,129,130,131]. Because of its important roles in replication and the life cycle of the virus, we can observe how N disorder can easily affect virulence and infectivity along with M disorder, as greater N disorder promotes more efficient protein–protein/RNA/lipid/carbohydrate recognition [16,30,31,32,33,132].

We have attempted to show that SDMs are highly reproducible and can account for many of the properties and manifestations of SARS-CoV-2-related viruses and COVID-19. Such reproducibilities should not be surprising, as M and N are the most abundant proteins in the virion and cell, respectively, and they play major roles in the life cycle of the virus, especially regarding its replication [105,106,107,108]. Nevertheless, we need to keep in mind that M and N are, of course, not the only viral proteins in CoVs. While they play major roles in the replication of the virus, other proteins also play roles. For instance, N binds to NSP2 before the transcription of viral RNA takes place [129]. Since it involves other proteins, we should therefore expect limitations and potentials in N and M, similarly to what we have observed in S. One such example is the use of virulence–inner SDM to compare distant viruses within a family. This is the case when the virus’s proteins are essentially very different in sequence and use different receptors. Other examples of the limitations of SDMs can be detected in the results of certain animal and viral titration studies. A discussion with further details can be provided in the next subsection.

### 5.10. A More Accurate Understanding of S Comes with the Study of Other Proteins

The above sections do not show that S does not play any role in infectivity and virulence; rather, they show that S does not play a role in infectivity and virulence in the manner that many scientists have envisaged. To be able to gain a more accurate understanding of the real role of S, a better understanding of the role of other viral proteins, especially the more abundant major proteins, is necessary.

We have presented evidence to show that S is not able to account for the differences in virulence and infectivity between SARS-CoV-1 and SARS-CoV-2. We have also seen that the viral titers of the two viruses are different, with higher titers, implying higher virulence in the case of SARS-CoV-1. While the presence and absence of FCS in SARS-CoV-2 and SARS-CoV-1, respectively, could explain the greater virulence of SARS-CoV-1, it does not explain the greater virulence of SARS-CoV-2. The SDMs using N and M provide a comprehensive and coherent explanation that is more reproducible. The SDMs are also able to explain the differences among COVID-19 viruses, even if they are more complex. Much of the problem lies in the fact that many of the viruses have yet to be observed to spread to the human population, and their actual pathogenicity and infectivity among humans cannot be accurately determined. For these phenomena, we must rely on animal models, the use of which is tricky and requires extrapolation. Incorrect inferences based on incorrect extrapolations and assumptions can easily be made, as we have observed in the disastrous pre-clinical studies of thalidomide using mice, rats, and rabbits [133].

In the case of COVID-19, it was believed that the FCS is responsible for infectiousness and virulence based on an animal model, in which infected and uninfected ferrets were caged together. One group had a few ferrets infected with wild-type SARS-CoV-2 (WT), whereas the other ferrets were infected with a mutated strain without FCS. The result was that the WT did infect some of the uninfected ferrets, whereas those infected with the FCS-mutant did not infect others [134]. Based on this experiment, it is easy to conclude that FCS is responsible for high transmissibility. A picture that contradicts this conclusion was obtained when similar experiments were performed using pangolin-CoVs and Omicron.

In various independent laboratories, uninfected Syrian hamsters were easily infected by those infected with pangolin-CoVs (Pang2017 and Pang2019) when caged together by contact, although this did not occur as easily as via aerosol [71,72,77]. Similar experiments using Omicron yielded comparable results [72,73,112,134]. Furthermore, it was observed that Wuhan-Hu-1 is more easily spread via aerosol, whereas pangolin-CoVs and, to some extent, Omicron were not as easily transmitted, even though the latter have been shown to spread easily clinically. Keeping in mind that pangolin-CoVs do not have FCS but Omicron does, these experiments highlight that the issue is more complex than it seems, and it is easy to come to the wrong conclusion without a more complete picture. Adding to the confusion, why does Pang2019 exhibit more virulence than Wuhan-Hu-1, which is not observed in Pang2017 and Omicron?

A more complete and reproducible answer can be found if the analysis takes the various roles of N, M, and S into consideration. It is obvious that S plays some role in the infectivity of the virus, but it is a mistake to assume that this role is as overreaching as suggested by some. SDMs explain that all thus far known variants are highly infectious, as COVID-19 patients shed large quantities of the virus because its hard outer shell M helps prevent virion disintegration caused by salivary and mucosal enzymes before being expelled by the body. While the abnormally hard M ensures a certain high level of spread, higher N PID (i.e., higher levels of N disorder) results in greater levels of viral replication and viral load (the mechanism has been explained in Section 5.9), especially in vital organs such as the lungs, which could increase the virulence of the virus and the amount of viral particles expunged from the body, even if the quantity of viral shedding is already high. This is exactly the case with Wuhan-Hu-1 and Omicron. Omicron has a relatively low N disorder (PID ~43%) and is therefore milder than the other variants, but it is also infectious, as it also exhibits low M PIDs (~5.4–5.9). Wuhan-Hu-1, on the other hand, is both highly infectious and more virulent, as its M and N PIDs are low and relatively high, respectively. The aforementioned animal model suggests that Wuhan-Hu-1 is relatively more infectious than Omicron. Since both SDMs and clinical studies suggest high infectiousness in the two viruses, the experiment can only be consistent with SDMs and clinical studies if the infectiousness of the two viruses is only relative in differences [79,80,81,112,134]. We have to be careful when using animal models since extrapolation is necessary when we attempt to link the results to human infections [135,136].

Furthermore, human infectivity tells only part of the story in the evolution of SARS-CoV-2 and its ancestral strain. We must also keep in mind the infectivity of SARS-CoV-2 and its relatives in different animal hosts. Furthermore, we must pay attention to data pertaining to virulence, including viral titration and cell damage. As virulence can be linked to infectivity using the SDMs and MCC, as shown in Section 5.4, Section 5.5 and Section 5.6, these factors are important when it comes to the interpretation of the results of animal models involving infectivity. We will also observe, in the next section, that the best understanding of the results comes from our knowledge of S, N, and M.

### 5.11. Biological Implications of a More Rigid M

We have seen in Figure 2 that all SARS-CoV-2-related viruses have an extraordinarily hard outer M. Nearly all CoVs have PIDs of above 8. The exceptions are CoVs associated with burrowing animals. CoVs closely related to SARS-CoV-2 have M PIDs between 4% and 6.3% (Table 2). The differences between burrowing and non-burrowing seem small, even if statistically significant. A clearer understanding is obtained, however, if we consider the functions and virion physiology related to M. M is the most abundant viral protein that spans the entire virion. Therefore, even with a few mutations that lower the disorder of M, the rigidity of the SARS-CoV-2 membrane is likely be strengthened substantially by the sheer abundance of M. This is especially true when the virus is in its native state and when M plays the essential role of protecting the virion from external attacks.

A question that arises is as follows: If SARS-CoV-2 M is more rigid, how is it able to perform its other functions that require conformational changes, such as viral entry and protein assembly? If we look at Table 2, we observe that the minimum M PID seen in all CoVs is around 4%. Obviously, this is the minimum level of disorder that is needed for M to have sufficient flexibility in order to perform its other roles that require conformational change. An M PID of 0% theoretically means that the protein has no room for conformation changes. Therefore, an M PID of 0% means that the virus is not functional. The data in Table 2 and Figure 2 seem to reinforce this idea due to the existence of the 4% minimum cutoff point. While a lower M PID offers greater protection to the virus in its native state, it could also imply that M could have less efficiency in undergoing conformational changes. We do not, however, know how substantial this impact is on the efficiency of conformational changes that are necessary for processes such as viral entry and protein assembly. Extrapolating from the efficiency of SARS-CoV-2 (M PID ~6.3%) with respect to replication, it is likely not substantial; however, once again, this is only an extrapolation that must be confirmed by experimental results.

## 6. Potentials and Limitations of M and N in SDMs

### 6.1. A Comparative Analysis of SARS-CoV-2, Pangolin-CoVs, and Laotian Bat-CoV Experiments Using S, N, and M

A more comprehensive analysis can be accomplished when examining M, N, and S in the experimental data for SARS-CoV-2, bat-CoVs, and pangolin-CoVs. We have observed that all COVID-19-related viruses are potentially infectious because of their abnormally hard M. We have also observed that Pang2019, BANAL (Laotian bat-CoV), and Wuhan-Hu-1 are all potentially virulent because of their high N PIDs (~48%), whereas Pang2017 and Omicron are attenuated as a result of their relatively low N PIDs (~44%). These have been largely reproduced. Cells infected with Pang2017 or Omicron have signs of lower viral growth and cytopathic effects [55,70]. Similarly, animal models have exhibited less severity. In contrast, however, viral titers of cells infected by Pang2019 [76,77,78] and Wuhan-Hu-1 [71,75] are higher than those infected by Pang2017 or Omicron [55,70,71,72], just as predicted by the SDMs. In contrast, mice were observed to be severely sickened by Pang2019 [77,78]. We need to keep in mind that this trend is true even though FCS is found only in SARS-CoV-2, including Omicron. Therefore, the stark differences in the results of various experiments can only be accounted for when the roles of N and M are considered.

### 6.2. Evidence of the Potentials and Limitations of S: Viral Entry and Replication

The data for the Laotian bat-CoVs present an enigma [50,51]. It was shown that BANAL S binds to ACE2 in a different manner from SARS-CoV-2, even as the BANAL viruses bind efficiently to human cells. Ironically, even though the N PID of BANAL is similar to those of Wuhan-Hu-1 and Pang2019, no severe disease was detected in mice infected by BANAL-236. This seemed to be inconsistent with what SDMs predicted until the data were studied very carefully. If we inspect the viral titration data, we can observe that the viral titers in VERO-E6 cells are high and comparable to Wuhan-Hu-1, but this is not the case in CALU-3 cells, where the difference is larger; it should also be kept in mind that a VERO-E6 cell is of kidney origin, whereas CALU cells are respiratory. This implies that BANAL-236 is more adapted to kidney cells than respiratory ones, presumably because of its S structure. We also need to keep in mind that the Laotian bat-CoV S protein binds to ACE-2 in a different manner [51].

While this serves as evidence that S does play a role in infectivity, it does not show that SDM results are wrong or not reproducible. Instead, this phenomenon points to the correct manner by which SDMs should be interpreted. Firstly, the experimental data remind us that SDMs predict *potential* virulence—not necessarily actual virulence—and this potential virulence arises from the high viral load in at least one organ, which is populated with cells that the virus can enter more easily. A second lesson to be learned from the data is that SDMs predict virulence in general—not just humans—since N PID correlates best with the highest viral titers among various cell types [55,70,71,72,73,74,75], and high viral loads are associated with organ failures. While human COVID-19 fatality is mainly associated with pulmonary (lung) failures, this may not be necessarily so for other animals. Therefore, SDMs are predicting potential virulence in general—not only in humans.

If there is evidence that S does play some role in infectivity and virulence via viral loads, the question then becomes the following: How easily does S adapt to bind more efficiently? It would seem that greater S fitness may be more easily acquired than many believe. Experiments have shown that COVID-19 viruses acquire a greater ability to infect the lungs after passing to humanized mice several times [133,137,138,139]. Furthermore, a closer comparison of the two may provide clues. Given the fact that both BANAL-236 and Pang2019 have high N PIDs, why has Pang2019 been shown to be virulent to humanized mice, unlike BANAL-236? Evidently, Pang2019 S is more adapted than BANAL-236. To understand why this is the case, we need to look at the evolutionary differences between the two viruses. If we look at Figure 5B, which shows a phylogenetic tree using M, we observe that Pang2019 is more closely related to SARS-CoV-2 than BANAL-236. This implies that Pang2019 was exposed to a similar range of hosts as Wuhan-Hu-1. What is also remarkable is that both BANAL-236 and Pangolin-CoVs, unlike SARS-CoV-2, do not have FCS. It is likely that Pang2019 split off from SARS-CoV-2 to mainly infect pangolins, and as a result, it lost its FCS while still maintaining much of its S structure. This may also have implications for Peacock et al.’s FCS-mutant experiment [111]. It is possible that the FCS-deficient S compensates for its deficit by binding to ACE-2 in a different manner over a longer period, as is the case with Pang2019. Moreover, as we will observe, it is easy for S to quickly adapt to the ACE-2 of a particular species or cell type in a laboratory.

It is evident that S must be at least sufficiently adapted to ACE-2 in order to even be infectious, but in order for SARS-CoV-2 to have any sustained infectivity or virulence, other factors must also come into play, especially the roles of M and N, as observed in the experimental and clinical evidence pertaining to SARS-CoV-1/2. The question then becomes the following: How difficult is it for S to gain sufficient adaptation to respiratory cells? The earlier section argues that it may not be very difficult. Several studies have shown that COVID-19 viruses can easily and quickly adapt to human respiratory cells in the laboratory [133,137,138,139]. Acquiring abnormally hard M that provides for potentially high infectivity, on the other hand, may not be as easy. It is relatively easy to identify CoVs that efficiently bind to the S protein of respiratory cells [100,108,115], possess an FCS [100,108,115], or exhibit easily adaptable S [107,131,136]. In contrast, CoVs with an abnormally hard M are rare, as most CoVs are not intimately associated with a burrowing animal, unlike COVID-19-related viruses [4,16,17,18,19,20,53,54,55,56].

### 6.3. Greater Model Reproducibility and Reliability Come When More Proteins Are Considered

The greater reliability and reproducibility of SDMs arise from the fact that they consider the disorder of two major proteins, M and N, which are most abundant in the virion and infected cell, respectively. In fact, SDMs become unreliable if M or N is omitted from consideration. SDMs are reliable and reproducible in most cases, except on certain occasions when the role of S must be taken into serious consideration, as observed in the case of the Laotian bat-CoVs. For this reason, we cannot dismiss S or any other proteins as unimportant. In fact, as we have shown, SDMs become even more reliable and reproducible when S (or other protein) is taken into consideration. This trend is consistent with what we know about the biology of viral replication. Viral replication involves multiple proteins, even if there are proteins that play more major roles than others.

### 6.4. Limitations and Potentials of N, M, and SDMs

We have already touched on the limitation of SDMs. We have seen that it cannot account for some experimental results conducted using BANAL (Laotian bat-CoVs). They are, for example, unable to account for the differences in the viral titrations of BANAL-236 and SARS-CoV-2 with respect to different cell types (e.g., CALU, COCA, Vero-E6) [51,52,73]. The significance of the differences, however, needs further investigation, as other researchers have shown that SARS-CoV-2 becomes more efficient in replication as several passages are made in each cell type. Nevertheless, this points to the role of S, which is a limitation of SDMs. Currently, SDMs are involved in viral shell proteins, which, in the case of CoVs, are the N and M proteins. Given that SARS-CoV-2 has 29 viral proteins and many are also involved in the replication process [105,106,107,108], there will be limitations if only one or two proteins are used to study infectivity, virulence, or long COVID. This is, of course, the case in SDMs, which currently use only M and N. In fact, as already mentioned, without either M or N, SDMs would be of limited reproducibility and reliability, and they would have much difficulty explaining the underlying cause of infectivity and virulence.

Ironically, the question is then the following: How can M and N account for infectivity and virulence with even a decent level of reproducibility and reliability when the limitation of using only two proteins is considered? The answer could lie in two factors. The important roles of M and N, as observed above, are likely to be partly responsible. Secondly, a hint of the overwhelming importance of M and N can be observed via the massive abundance of proteins, which is not seen in other COVID-19 viral proteins. This could also reinforce the idea that the two proteins play greater major roles in the functioning of the virus. While the focus of this review is on SDMs and N and M proteins, we do not dismiss the importance of S or any other viral proteins. This review, however, attempts to underscore the importance of M and N in infectivity and virulence, as exemplified by the examples shown in Figure 6 and Figure 7. The results in Figure 6 and Figure 7 do not invalidate the role of S—as Hui et al. attempted to demonstrate—but suggest that the currently understudied roles of M and N could even overshadow that of S with respect to infectivity, virulence, and potentially long COVID. Of course, SDMs would become more reproducible and reliable if more viral proteins, such as S and NSP7, are considered in the models. However, currently, SDMs have not reached the stage where the roles of other proteins can be incorporated.

## 7. SDMs Hint at a Novel Immune Evasion Strategy Used by SARS-CoV-2 in Long COVID

### 7.1. The Long COVID Enigma and Pangolin Footprints

One unusual characteristic that is often found among some COVID-19 patients is long COVID, which is when symptoms persist for weeks, months, or even years after the initial infection [57,58,109,110]. While the cause of long COVID remains largely a mystery, SDMs offer the most logical and plausible explanation yet. We have seen that all COVID-19-related viruses have extraordinarily hard outer shells (M) that are not found in any CoVs, except those associated with burrowing animals. We have also observed how SDMs explain that the hard M allows for greater COVID-19 infectiousness by providing more resistance to mucosal and salivary antimicrobial enzymes, often without the greater virulence that is associated with greater N disorder [18,19,20,52,55,56]. Similarly to how a hard M provides resistance to antimicrobial enzymes, it will almost certainly also provide resistance to other destructive mechanisms offered by other aspects of the host immune system. This is possible as clinical studies have shown that large amounts of the virus tend to remain in the body even after months [109,110]. We will further explore this link via a further examination of the various known aspects of immunology related to this matter, as detailed in the following subsections.

### 7.2. Hard M Resistance to Virolysis by Phagocytes and a Complement System

In order to further examine the role of an abnormally hard M in long COVID, we need to look more closely at how the immune system eliminates invading foreign particles, especially viruses. Our knowledge of immunology helps focus our attention on proteins produced by the complement system and lysosome [138,139,140,141,142,143,144,145]. These enzymes are experimentally shown to damage viruses and viral membranes.

Complement proteins are mainly produced by hepatocytes in the liver, even though they are secreted by monocytes, macrophages, and epithelial cells in the intestines. There are at least 30 types of complement proteins [142]. B and T cells, along with antibodies, can alert the complement system in the presence of foreign matter such as bacteria or virus. Complementary proteins assemble and bind to a protein at the targeted membrane, and the protein complex punches holes in the membrane. Apoptosis occurs in the case of bacteria or infected cells, whereas, in the case of a virus, this is referred to as virolysis. It is at this point in COVID-19 and long COVID that the hard M may make it difficult for the complement system to carry out its function, as the proteins are likely unable to penetrate the membrane since M spans the entire membrane that covers the virion.

### 7.3. Resistance to Virolysis Within a Phagocyte May Provide the Virus a Place to Dwell: Possible Reservoir

A second way that the immune system attempts to eliminate pathogens is to expose them to digestive enzymes [130,131,143]. Phagocytes, such as macrophages, will first engulf the microbe or microbial protein, and upon phagocytosis, the immune system will attempt to digest the particle via lysosomes [141,142,143,144]. The problem with this strategy, however, is that many pathogens are somehow resistant to the exposure of digestive enzymes and end up residing in the phagocytes [143,144]. An ongoing enigma pertaining to long COVID concerns the existence and location of a viral reservoir that persists long after the initial infection. A hard outer shell could prevent phagocytes from destroying the virus, allowing it to survive within these immune cells. The observation of an abnormally hard M, along with our knowledge of immunology, suggests that the first place to look at is none other than the phagocyte and, in particular, macrophage itself, and current research supports this. Huot et al. [146] found the presence of SARS-CoV-2 in the lungs of patients with symptoms of long COVID, whereas several other research groups have found its presence in organs and tissues throughout the body [109,110], which is consistent with the fact that phagocytes can be found in nearly all organs in the body [142].

The nature of SARS-CoV-2 should not be confused with that of other viruses, such as HIV and HSV. The fact that SARS-CoV-2 has the hardest outer shell—not only among CoVs but also among all viruses—is a tell-tale sign that the virus is of a different nature than other viruses such as HSV and HIV-2, which are known to hide in locations such as the brain only to present themselves later [108]. HIV and HSV-2 have one of the most disordered outer shells among viruses, unlike SARS-CoV-2. Their highly disordered outer shells help them penetrate and hide in organs, as greater disorder allows for more efficient protein–protein binding. SARS-CoV-2, on the other hand, has one of the hardest outer shells. If it does not have the benefit of a disordered outer shell, how does it hide? The answer lies in the mechanism of phagocytosis described above. There is research showing that inflammatory responses may be responsible for long COVID. A hard M and inflammatory responses, such as those caused by cytokines, are not mutually exclusive [145,146,147]. Due to the extraordinary hardness of M, SARS-CoV-2 can hide in phagocytes only to appear whenever the opportunity arises. Whenever the virus keeps reappearing, the immune system could respond in a manner that could result in inflammation caused by cytokines.

### 7.4. Granzymes: A Suspected Mechanism of M Resistance

While the exposure of destructive enzymes has been shown to act against viruses in the complement system and macrophages, there are also other destructive enzymes available in the immune system. These involve a family of enzymes known as granzymes. Upon entry of the virus, the immune system will initiate a variety of defensive actions that include cytotoxic T-cells and natural killer (NK) cells, which secrete substances that could potentially damage viral particles [139,140,141,142]. These cells secrete granzymes in response to a foreign invader. One of the granzymes is perforin, which binds to plasma membranes and punches holes that allow other granzymes to enter, causing further damage to the bacterium or infected cell [148]. While current experimental evidence has shown this to occur in the membranes of bacteria and infected cells, there is currently no evidence that it damages viral membranes. Nevertheless, given the known biochemical capabilities of perforin, perforin can potentially damage the virion in the same manner, since most animal viruses, including SARS-CoV-2, have a protective outer membrane layer. In any case, perforin causes lysis in infected cells, thereby allowing viral particles to be exposed to the complement system and macrophages. It is also known that, while T-cells and NK cells secrete perforin, macrophages secrete perforin-2 (PFN2), which is similar to perforin, but it remains unclear whether PFN2 affects viral membranes directly [146,148].

### 7.5. Uniqueness of COVID-19 Strategy of Immune Evasion in Long COVID

There are many mysteries involving long COVID that physicians and scientists are still struggling to solve with respect to creating better treatments. How does SARS-CoV-2 induce long COVID? What are the mechanisms? Is there a reservoir? If so, where is it? As we have observed, SDMs offer specific answers to these questions. While N disorder modulates the amount of virus replicated, especially in vital organs, the unusually hard M provides resistance to antimicrobial enzymes. Therefore, the immune evasion strategy used by SARS-CoV-2 is drastically different from that of viruses such as HIV, HSV, and HCV [4,16,17,18,19,20,65,66,67], which have high disorder in the other shell that allows such viruses to hide in organs. Not only has no comparable high disorder been detected in SARS-CoV-2, but the virus has one of the hardest outer shells among viruses—not only among CoVs. This is why phagocytes, such as macrophages, can serve as a refuge, allowing the virus to dwell and hide in them. Indeed, one laboratory showed the presence of the virus in the lung alveolar macrophages of patients who tested negative for the virus in the upper respiratory system [147]. What is even more puzzling is that several other laboratories have observed the presence of the virus in many organs of long COVID patients [110]. This raises the following question: Where is the reservoir? Again, if macrophages and phagocytes are the reservoir, the virus will be present in various organs since phagocytes are found in nearly every organ in the body.

### 7.6. Long COVID, Long SARS, and S

Long COVID has been linked to the activation of the immune system via interferons (IFN-γ) and natural killer T-cells (NK cells), which results in inflammation [145,146]. Many scientists point to S as the main underlying cause of the activation. While this postulation is very interesting and plausible, it raises more questions than it answers. For instance, it does not tell us the reason why some people experience long COVID while others do not. It does not tell us whether long COVID arises from a reservoir, and if so, what the source of the reservoir, i.e., the hiding place of the virus, might be. We also need to remember that SARS-CoV-1 has a CoV S protein as well. Because the two viruses are relatively closely related (80%), it is likely the S of both viruses are functionally similar, including their ability to activate the immune system in similar ways. A conundrum is, however, observed upon an examination of the differences between long COVID and long SARS. One stark difference is the fact that long SARS is usually associated with the severe manifestation of the disease, whereas long COVID could present itself even with mild symptoms {64]. How could there be such a disparity if the S proteins of the two viruses are likely to be structurally similar and when it has been shown that S activates the immune system [145,146], which is likely to cause long COVID? SDMs offer a novel explanation. SDMs have observed that SARS-CoV-2 has a much harder M than that of SARS-CoV-1; as a result, the virus can resist immune enzymes and hide in the phagocyte in the case of COVID-19. We are, therefore, likely to obtain a better picture when we consider the roles of M, N, and S, keeping in mind that N also plays a role, as greater N disorder could permit the greater production of viral particles—even in the event of long COVID.

## 8. Summary and Conclusions

### 8.1. SDMs: Coherent Links Among Virulence, Infectivity, and Long COVID

This review focuses on three closely related models with respect to COVID-19. The models use intrinsic protein disorder to link N and M proteins to infectivity, virulence, and potentially long COVID. While the three models—shell disorder models (SDMs) [4]—were initially computationally created using empirical AI molecular techniques based on intrinsic protein disorder, experimental and clinical data have been examined for the reproducibility and reliability of SDMs. Interestingly, SDMs are uniquely able to link the major cause of infectivity, virulence, and potentially long COVID under one coherent concept of intrinsic protein disorder. An example of the reliability and reproducibility of SDMS is observed when SDMs were able to provide a novel and coherent explanation of the differences in virulence and infectivity between SARS-CoV-1 and SARS-CoV-2, which has been clinically shown to induce much greater shedding of infectious particles in patients. Curiously, all known SARS-CoV-2-related viruses, excluding SARS-CoV-1, have an abnormally hard outer shell (low M disorder), which is associated with burrowing animals, such as rabbits and pangolins. Evidence of a unique molecular and evolutionary relationship (“pangolin footprints”) between pangolins and COVID-19 has been examined. SDMs suggests that this hard outer shell is responsible for the high infectivity of COVID-19 and, potentially, long COVID, as the hard M provides resistance to the antimicrobial enzymes found in immune and respiratory systems. The implications could provide clues towards further research involving long COVID and infectivity, including the search for possible reservoirs among phagocytes. This also explains clinical observations of the persistence of the virus throughout the body months after infection. In the case of virulence, greater disorder in N contributes to more rapid replication by providing more efficient protein–protein binding. As a result, N disorder correlates with viral titers and, therefore, virulence; moreover, to a limited extent, it is also correlated with infectivity. As S (spike protein) is currently yet to be part of SDMs, S is mentioned sparingly as part of a discussion involving the potentials and limitations of SDMs.

### 8.2. Unusual Characteristics in SARS-CoV-2 and the Clinical Manifestations That Arise from Its Evolution

The phrase “pangolin footprints” refers to a set of molecular signatures that were left behind by the intimate evolutionary interactions COVID-19 ancestral strains had with pangolins. These signatures—which involve the abnormally hard M and the trend towards lower N disorder—arose from the pangolins’ burrowing habit, which entails a harder outer and inner viral shell to facilitate oral–fecal transmission via buried feces. These features—or pangolin footprints—are clinically manifested in the symptoms, infectiousness, and attenuation/virulence of COVID-19. A review of the current knowledge of immunology indicates that these features are also manifested as long COVID. The abnormally hard M, which facilitates greater infectiousness by being resistant to antimicrobial enzymes in the saliva and mucus, can also resist elimination attempts by the immune system, thus resulting in long COVID.

### 8.3. Clues Pointing to Pangolin Footprints in the Evolution of SARS-CoV-2

A unique and highly unusual property that involves all examined SARS-CoV-2-related viruses is the hard outer shell (low M PID). This property is very rarely found even among the CoV family. What is the evolutionary origin of this peculiar characteristic? A retrospective search of our disorder database of CoVs provided us with a clue when it was found that very few CoVs have such exceptionally hard M, and those found were associated with a burrowing animal, such as rabbits. Furthermore, it can be observed that phylogenetic trees using M have a much closer relationship to SARS-CoV-2 than previous phylogenetic trees have shown. How can this be possible when RaTG13 exhibits 96.4% similarity to SARS-CoV-2, whereas pangolin-CoVs exhibit approximately only 90% similarity to SARS-CoV-2? What is even more puzzling is that one of our trees shows that Omicron has a closer relationship to pangolin-CoVs than to other variants. The answer is that phylogenetic algorithms do not handle recombinations well, and M may be the best choice for phylogenetic studies since M is abnormally structured (low disorder), which means that it is likely to be highly conserved. Secondly, many scientists have searched for an intermediary animal host for SARS-CoV-2, but so far without success. It has obviously not occurred to many scientists that there is no intermediary animal host because the ancestral virus has entered and re-entered the human population and other animal populations over a long period of time, with the primary or secondary reservoir being pangolins. This may explain why all SARS-CoV-2-related virus have unusually hard M proteins and why SARS-CoV-2 is highly infectious, not only to humans but also to a wide range of animals. It takes time for the virus to become highly adapted to such a wide variety of animals. All these phenomena present clues of a more unique evolutionary relationship between pangolins and SARS-CoV-2 that needs to be further researched, as there are hints that the resulting characteristics are related to the behaviors and clinical manifestations of the virus.

### 8.4. Long COVID

As already mentioned above, while current research shows that S is definitely involved in the onset of long COVID [145,146], S does not offer a plausible hypothesis for the underlying cause of long COVID, as the source of the virus reservoir is not addressed. The underlying cause of long COVID has thus far remained a complete mystery, which is a great hindrance to the search for more effective treatments. M and, to a much smaller extent, N proteins via SDMS, however, offer an elaborate and highly plausible explanation using our current knowledge of immunology with respect to the cause of long COVID: The hard outer shell of the virus likely makes it difficult for phagocytes, T-cells, and other entities to eliminate it. As a result, there may be plenty of opportunities for the virus to dwell in phagocytes, which are a likely reservoir.

### 8.5. Clues for Further Research

We have seen that SDMs offer a novel coherent theory that links virulence and infectivity with N and M via intrinsic protein disorder. Experimental and computational evidence of the reliability and reproducibility [128,149] of SDMs as applied to COVID-19 was examined. We have attempted to show that SDMs using N and M are, by and large, reproducible when experimental and clinical data—especially from other laboratories—are scrutinized. The reason for the reproducibility and reliability of M and N can be partially traced to the abundance of the major proteins and their important roles in the replication process. The role of M in infectivity is based on the observation of an abnormally hard SARS-CoV-2 M using AI. Interestingly, the phylogenetic study of SARS-CoV-2-related viruses points to a closer relationship between pangolin-CoVs and SARS-CoV-2. This has not been shown in any other phylogenetic study using other proteins or entire genomes. While evidence of the reproducibility and reliability of SDMs as applied to COVID-19 using N and M has been presented, further experimental and clinical research is needed to re-affirm their reproducibility and reliability [128,149].

Even though M and N are the most abundant proteins in the virion and cell, respectively, there are 29 CoV viral proteins, of which many are also involved in replication and virulence. For this reason, we have tried to show that many of the limitations of COVID-19 SDMs arise from this fact. A solution would be to include more proteins, such as S and NSP7. This is where further research could help, since current SDMs only incorporate N and M.

While we have argued, using the existing framework of SDMs and current immunological knowledge, that SDMs offer compelling insights into long COVID, their application to this condition is still in its infancy, even if there is evidence that the virus can be detected in the blood and throughout the body, as predicted by SDMs. Given that long COVID is still largely a mystery and an important medical issue, it therefore imperative that long COVID is and continues to be further investigated into using novel tools such as SDMs. Further experimental and clinical studies are therefore necessary, given the clues presented by SDMs.

The clinical implications presented here are numerous. If the virus is hiding in phagocytes only to present itself intermittently, therapeutic strategies, such as antiviral drugs and vaccines, can be tailored to control the sudden release of the virus from its reservoir. Furthermore, SDMs provide novel strategies for vaccine development—not only for SARS-CoV-2 but also other viruses. Moreover, the concept involving virulence and infectivity can be used to monitor the progress of the current COVID-19 pandemic. Thus far, the trend in newer variants is moving towards consistently hard M proteins and lowering N disorder. If this trend continues, SDMs predict that COVID-19 will persist, maintaining high infectivity due to its hard M while presenting milder symptoms arising from its lower M PIDs. In addition, SDMs provide a strategy for detecting future potential pandemics—not only for CoVs but also other viruses.

## Figures and Tables

**Table 1:** Three shell disorder models (SDMs). SDMs were developed using the principles of intrinsic protein disorder applied to viral shell proteins.

Year of First Publication	Shell Disorder Model	Details
2008	Viral Shapeshifter Model (Parent)	Disorder of shell proteins was measured for a wide variety of viruses. Only very few viruses have been found to have unusually high disorder in the outer shell (HIV-1, HCV, and HSV). There is no effective vaccine yet found for the three viruses.
2012	CoV Transmission SDM	Links between modes of transmission (fecal, oral, and respiratory) and N and M disorder were found.
2015	Virulence–Inner Shell Disorder Model	Strong correlation between inner shell disorder and virulence of a wide variety of viruses, including DENV, EBOV, NiV, and SARS-CoV-1/2.

**Table 2:** N/M PIDs and sequence similarities of COVID-19-related CoVs, with SARS-CoV-1 and non-SARS-CoV-2-related bat-CoVs as references [55]. It should be noted that BA1 was the initial Omicron. At least two Delta subvariants have been detected by SDMs.

Coronavirus	Sequence Similarity M (%) ^a^	M PID (%)	Accession: UniProt (U); GenBank (G)	Sequence Similarity N (%) ^b^	N PID (%)	Accession UniProt (U); GenBank (G)
SARS-CoV-1	90.5	8.6	P59596(U)	90.5	50.2	P59595(U)
Civet-SARS-CoV	90.1	8.6	Q3ZTE9(U)	90.01	49.1	Q3ZTE4(U)
Laotian Bat-CoV	-	6.0 + 0.2	-	-	48.3 + 0.2	-
[Banal-52]	98.7	6.3	UAY13220.1	99.3	48.2	UAY13225.1
[Banal-103]	98.7	5.9	UAY13232.1	99.1	48.5	UAY13257.1
[Banal-236]	99.1	4.1	UAY13256.1	99.3	48.5	UAY1326.1
Pangolin-CoV	-	5.6 + 0.9	-	-	46.6 + 1.6	-
2019	98.2	6.3	QIG55948(G)	98	48.7	QIG55953(G)
2018	97.7	4.5	QIQ54051(G)	93.8	46.3	QIQ54056(G)
2017	98.2	5.9	QIA48617(G)	94	44.9	**QIA48630(G)**
				93.32	46.5	QIA48656(G)
SARS-CoV-2						
Wuhan-Hu-1	100	5.9	YP009724393(G)	100	48.2	YP009724397(G)
Delta		5.9 + 0.01			47.1 + 0.5	
Delta1	99.1	5.9	QUX81285(G)		46.8	QYM89997(G)
Delta2	99.1	5.9	QUX81285(G)	99.1	47.5	QYM89845(G)
Omicron	-	5.7 + 0.4	-	-	44.5 + 0.4	-
Omicron BA1	98.7	5.4	UFO59282(G)	98.6	44.8	UFO692871(G)
Omicron XBB	99.1	5.9	WBI50320(G)	98.2	44.2	WIL50325
Bat-CoV		11.2 + 15			47.7 + 0.9	
RATG13	99.6	4.1	QHR63303(G)	99.1	48.5	QHR63308(G)
Bat 512	35.5	15.3	Q0Q463(U)	29.4	46.5	Q0Q462(U)
HKU3	91	7.7	Q3LZX9(U)	89.6	48	Q3LZX4(U)
HKU4	42.7	16.4	A3EXA0(U)	51.1	48.5	A3EXA1(U)
HKU5	44.7	11.8	A3EXD6(U)	47.9	47.1	A3EXD7(U)

^a^ Sequence similarity of the M proteins with Wuhan-Hu-1 as the reference virus (i.e., 100% identity). In this column, we observe that the M protein of SARS-CoV-2 exhibits 90.5% sequence similarity to the M protein of SARS-CoV-1. This contrasts with the 89% genome-wide sequence similarity of SARS-CoV-1/2. Similarly, RaTG13 exhibits 99.6% M similarity to SARS-CoV-2, in contrast to the genome-wide 96.4% similarity. This supports the idea that M may be the most conserved CoV protein among all SARS-CoV-2-related viruses, as implied by the abnormal M hardness. ^b^ Sequence similarity of the N proteins with Wuhan-Hu-1 as the reference virus (i.e., 100% identity). In this column, we observe that the N protein of SARS-CoV-2 exhibits 90.5% sequence similarity to the N protein of SARS-CoV-1.

## Data Availability

Not applicable.

## Abbreviations

COVID-19	coronavirus disease
CoV	coronavirus
SARS	severe acute respiratory syndrome
HCoV	human coronavirus
SDM	shell disorder model
PONDR^®^:-VLXT	predictor of natural disordered regions using VLXT
PID	percentage of intrinsic disorder (number of disordered residues divided by the total number of residues)
Pang2017	SARS-CoV-2-related pangolin-CoV isolated in 2017
Pang2019	Pangolin-CoV isolated in 2019
N	nucleocapsid protein
M	membrane protein
S	spike protein
SARS-CoV-2, BANAL	SARS-CoV-2-related Bat-CoVs found in Laos
NL63	a common HCoV
RaTG13	a SARS-CoV-2-related bat-CoV discovered in Yunnan
AI	artificial intelligence
EBOV	Ebola virus
NiV	Nipah virus
DENV	dengue virus
HIV	human immunodeficiency virus
YFV	yellow fever virus
ZIKV	Zika virus
